# SAR11 ecotypes across ocean basins change with depth due to changes in light and oxygen

**DOI:** 10.1093/ismejo/wraf221

**Published:** 2025-10-08

**Authors:** Matthew D Hays, Clara A Fuchsman

**Affiliations:** Horn Point Laboratory, University of Maryland Center for Environmental Science, Cambridge MD 21613, United States; Horn Point Laboratory, University of Maryland Center for Environmental Science, Cambridge MD 21613, United States

**Keywords:** SAR11 ecotypes, mesopelagic, global ocean

## Abstract

SAR11 bacteria are ubiquitous and abundant heterotrophs that are important mediators of marine biogeochemical cycles. Within the SAR11 clade smaller ecotypes inhabit different ecological niches. Using metagenomic read placement onto a phylogenetic tree of RNA polymerase (*rpoB*), we were able to determine the distribution of different ecotypes both geographically and by depth. Our method avoids biases from the absence of quality sequenced genomes for deep SAR11 ecotypes. Depth profiles that range from the surface to the bathypelagic were analyzed at 30 stations in six ocean basins. In the euphotic zone, changes in the dominant primary producer from eukaryotic algae to cyanobacteria, did not cause the abundance of SAR11 to shift between stations. However, specific SAR11 ecotypes did correlate with eukaryotic phytoplankton (1a.3 and 1a.4) or picocyanobacteria (1b.2, 1b.4, and IIaB). In the lower euphotic and mesopelagic zones, group IIb.x was overwhelmingly the dominant species but group 1c was also present, and we found several new deep subecotypes of 1b. The shift between the surface SAR11 community, dominated by 1a and surface 1b subecotypes, and the mesopelagic ecotype groups, corresponded to the maximum decrease in the light-dependent proteorhodopsin*/rpoB* ratio, indicating that many deep ecotypes did not possess proteorhodopsin. This ecotype switch repeatedly corresponded to the maximum in Low Light I *Prochlorococcus*, leading to the hypothesis that changes in light motivates the ecotype switch. Environmentally abiotic factors like light and temperature appear to be determining factors in the SAR11 ecotype distribution throughout the global oceans.

## Introduction

Heterotrophic bacteria are a key component of the microbial loop, utilizing dissolved organic matter to produce bacterial biomass. The SAR11 order *Pelagibacterales* within *Alphaproteobacteria* are the most abundant marine bacteria in the oceans, estimated to account for 25% of planktonic cells and up to half of prokaryotic cells in the marine environment [1, 2]. They are small, free-living heterotrophs, with streamlined genomes, found throughout the world's oceans [1–3]. Their large surface area to volume ratio and a large periplasmic space with many high affinity ABC transporters allow SAR11 bacteria to consume simple dissolved organic C found at low concentrations in the environment [4, 5], but they are k strategists that cannot respond to sudden pulses of substrate [6]. Due to their streamlining, SAR11 cells need many exogenous compounds like amino acids, reduced organic S, and B_1_ vitamins [7–9]. The SAR11 clade breaks down phylogenetically into five well defined major groups with at least 10 major subclades [10, 11]. Single amino acid polymorphisms indicate that SAR11 ecotypes are ancient with high intrapopulation sequence diversity, implying a genetically stable population [12]. Some of these subclades are found to be specific to certain environmental regimes, changing with depth, oxygen, salinity and temperature/latitude [12–19]. SAR11 metabolic variations [4, 20, 21] have ecological implications that are magnified by the ubiquity of SAR11 in the marine environment.

Many heterotrophs, although they rely on previously fixed organic carbon for metabolic requirements, have proteorhodopsin genes that code for photoreceptor proteins which can have diverse biological functions in the environment [22–24]. Total proteorhodopsin abundance in the surface ocean has been linked to oligotrophy, and an inverse relationship has been found between proteorhodopsin and chlorophyll [25]. The theoretical cost benefit of proteorhodopsin suggests that at least 50 μmol photons m^−2^ s^−1^ of light is needed to counteract the cost of producing the required proteins [26]. Thus, a decrease in proteorhodopsin abundance with depth is theoretically predicted. In metagenomic and transcriptomic data from the subtropical North Pacific, total proteorhodopsin abundance and activity were greatly reduced at depth, though there was some evidence of transcription at 1000 m [27]. All known SAR11 cultures have proteorhodopsin [27], though present cultures are biased toward surface ecotypes [28]. Culture studies on a SAR11 clade endemic to the surface ocean (ecotype 1a.1) showed that, under normal growth conditions, no difference in growth rate or carbon fixation was seen with light, but under starvation conditions, cultures grown in light were able to maintain function and healthy morphology better than those grown in dark conditions [29]. At depth in the North Pacific, the types of proteorhodopsin differed from those in the euphotic zone, with one deep SAR11 proteorhodopsin weakly responding to light when cloned into *Escherichia coli* and some other deep proteorhodopsin proteins not responding to light at all [27]. It is unclear if deep SAR11 ecotypes possess light harvesting proteorhodopsin proteins.

In order to fully understand marine biogeochemical cycling and the fate of carbon in the oceans, greater consideration of the mesopelagic and bathypelagic ocean is necessary due to the volume of the oceans that these depth ranges occupy. The SAR11 subclades IIb, 1c, and Vb are thought to be mesopelagic subclades and subclade 1b has been found in both surface and subsurface waters [10]. It is debated whether SAR11 group V is actually a member of the SAR11 group or part of an outgroup within the *Alphaproteobacteria* [30, 31] but it was included in our analyses to encapsulate the entire possible breadth of the clade. Though high throughput culturing techniques for SAR11 continue to improve, the three deep subclades have not been cultured [28]. Single cell genomes of group Ic indicate a slightly larger genome and a difference in proportions of amino acids used, consistent with other bacteria adapted for meso and bathypelagic conditions [18]. However, the metabolism of the Ic clade was similar to previously cultured SAR11 ecotypes [18]. The other two deep subclades have not been genomically examined. Current understanding from metagenomic data is that SAR11 subclade Ic is the dominant clade below the euphotic zone [11, 15, 32]. However, these analyses are hampered by the lack of good genomes of the other deep SAR11 clades. A few single cell genomes (SAGs) for clade IIb exist from an oxygen deficient zone [15] (ODZ), and one partial metagenomic assembled genome (MAG) from the Arctic [16], but they are all quite incomplete and may not be representative of the clade.

Clades within SAR11 fit into distinct ecological niches in specific environmental conditions [11]. So far distinct ecotypes have been found in the high salinity Mediterranean [11], cold high latitudes [14, 16] and in anoxic ODZs [15, 32]. They are areas of the mesopelagic ocean that are naturally anoxic (<10 nM O_2_) and are critically important to the global nitrogen cycle [33, 34]. There are three main oceanic ODZs: the Eastern Tropical North Pacific oxygen deficient zone (ETNP ODZ) offshore from Mexico, the Eastern Tropical South Pacific oxygen deficient zone (ETSP ODZ) offshore from Peru and Chile, and the Arabian Sea in the Indian Ocean. In all ODZs, the anoxic core is surrounded by a transition shell of hypoxic waters [35]. Without oxygen, the microbial community relies on nitrate as an electron acceptor, sometimes reducing it all the way to N_2_ gas via denitrification, removing fixed N from the ocean [36, 37]. In oxic conditions, SAR11 bacteria utilize oxygen, but in anoxic waters they are predicted to be nitrate reducers but not N_2_ producing denitrifiers [15, 32]. SAR11 is >30% of the community in both the ETSP and ETNP, and is potentially the most abundant nitrate reducer in the ODZ [15, 18, 32], and SAR11 IIa.A and 1c ecotypes are particularly abundant in ODZs [15, 32]. Using Metagenome Assembled Genomes (MAGs) and SAGs, a member of the subclade IIa.A has been named as *Candidatus* Anoxipelagibacter denitrificans and found to be endemic to ODZs with the ability to use nitrate as an electron acceptor [32, 38]. In contrast, in the Antarctic and Arctic, where temperatures are extremely cold, the subclade Ia.1 is the dominant surface clade, which is attributed to cold adaptation [14, 16]. Additionally, polar specific groups also existed in both group IIa and IIb [16]. Within the subclade Ia.3, a genetically distinct lineage has been found to be particularly abundant in the salty Mediterranean [11]. Thus, distinct ecotypes have been found in a diversity of extreme conditions.

In the current common pipelines, SAR11 ecotypes are not properly determined in deep waters. In amplicon datasets, SAR11 ecotypes are generally determined at the group I, II, III, and IV level [39, 40], which is not detailed enough to separate surface and deep ecotypes. SAR11 ecotypes have been examined in metagenomes but have relied on read mapping techniques which require good reference genomes. Though these techniques are used to examine euphotic zone metagenomes [11, 41], they are not yet applicable to the deep ocean due to the lack of genomic representation of the deep ocean SAR11 clades. Attempts to examine SAR11 ecotypes in metagenomes from deep waters have demonstrably missed a large portion of the total SAR11 population, with the proportion of the community that was SAR11 being an order of magnitude lower in the metagenomic data compared to fluorescent *in situ* hybridization (FISH) counts [13].

The analysis uses metagenomic read placement on highly detailed phylogenetic trees of single genes [36, 42]. Our analysis places reads broadly on a tree that mixes both references and assembled metagenomic contigs, which allows for environmental diversity to be categorized and semi quantified. Use of this method with single copy core gene RNA polymerase (*rpoB*) allowed us to accurately place metagenomic read data into both surface and deep ecotypes and directly compare quantities of each subgroup in depth profiles throughout the water column at locations across the globe. We hypothesized that light was structuring both the SAR11 community and which members contained the proteorhodopsin gene.

Unfortunately, there was no available Photosynthetic Active Radiation (par) for the majority of the stations examined here. Therefore, we decided to utilize *Prochlorococcus* ecotypes as a proxy for light level. *Prochlorococcus* ecotypes change dramatically with depth with highlight ecotypes in the surface, Low Light I (LLI) in the middle of the euphotic zone at the deep chlorophyll maximum (DCM), Low Light II below the DCM and Low Light IV at the bottom of the euphotic zone [43–46]. DCMs are thought to occur at the balance between light and nutrients, with light being the ultimate control; e.g. at the Hawaii Ocean Time series (HOT) the DCM occurs at ~0.5 mol photon m^−2^ d^-1^ [47]. Thus, *Prochlorococcus* ecotypes allow us to judge the light levels experienced in metagenomic samples, and to address our hypothesis, without PAR data.

## Materials and Methods

**Figure 1:**
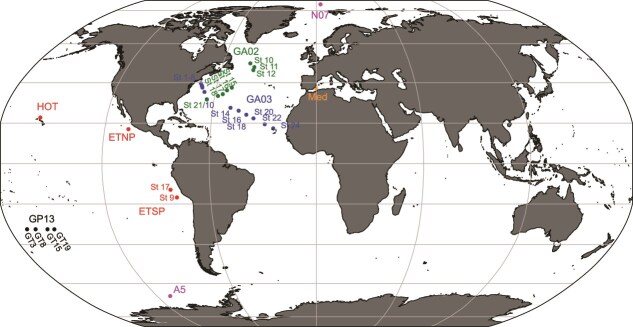
Map of stations included in analysis. In the North Atlantic, the stations in blue are from the GA03 transect and the stations in green are from the GA02 transect.

Metagenomic reads and contigs were downloaded from publicly available data across 30 stations and compared (Fig. 1). All depth profiles consist of metagenomes from four or more depths (average *n* = 8 depths). Stations were chosen to capture both the euphotic zone and the mesopelagic with the goal of capturing the transition between the two while also maximizing coverage across the different oceans. Study sites included multiple spots in the Pacific including samples from the HOT series in the North Pacific subtropical gyre from May (272), August (275), and November (278) of 2015 [48, 49] (Bioproject PRJNA352737), one station in the ETNP ODZ from April 2012 [36] (Bioproject PRJNA350692), two stations in the ETSP ODZ from July 2013 [50] (Bioproject PRJNA704804), four stations from Geotraces GP13 transect in the South Pacific subtropical gyre from June 2011 [51] (Bioproject PRJNA385854). Additionally we include one station in the Arctic in August 2017 [52] (8th Chinese Arctic Expedition), one station in the Amundsen Sea in Southern Ocean near Antarctica from March 2018 [52] (34th Chinese Antarctic Expedition) (Bioproject number PRJNA588686), 13 stations from the North Atlantic Geotraces East–West fall transect (GA03) from November/December 2011 [51] and seven stations from the North Atlantic Geotraces North–South spring transect (GA02) from May/June 2010 [51] (Bioproject PRJNA385854), and one station in the Mediterranean Sea from October 2015 [53] (BioProject PRJNA352798) (Fig. 1). All samples were collected on 0.2 μm filters. The Mediterranean samples were prefiltered through a 5 μm filter [53], but all other samples were not prefiltered. Most metadata can be found within the cited papers, but for Geotraces cruises (GA02, GA03, and GP13), CTD and nutrient data were downloaded from the British Oceanographic Data Centre (https://www.bodc.ac.uk/geotraces/) as part of the GEOTRACES 2021 [54] Intermediate Data Product (IDP2021). SRR numbers as well as available metadata for each station can be found in Table S1.

**Figure 2:**
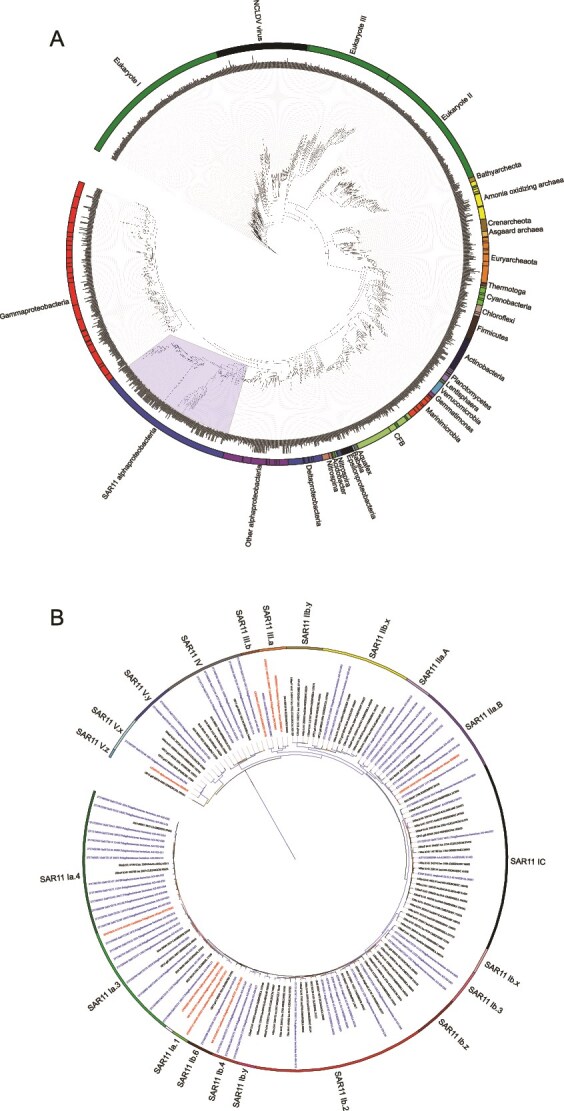
(A) An amino acid phylogenetic tree for RNA polymerase subunit beta. The blue shading encapsulates the SAR11 branch of the tree and is expanded and labeled in detail in B. (B) The SAR11 section of the tree is labeled with ecotypes. The blue sequence names are single cell references or MAG references and the red sequences are cultured. Taxa names on trees are very small and thus not intended to be read in print form.

The detailed protocol for creating phylogenetic trees and for placement of metagenomic reads on those trees is available at protocols.io [55]. We prepared three phylogenetic trees: (i) a bacterial and archaeal single copy core gene *rpoB* to examine SAR11 ecotypes, (ii) a photosynthesis PSII D2 polypeptide gene (*psbD*) tree to examine both *Prochlorococcus* ecotypes and the identity of eukaryotic algae, and (iii) a proteorhodopsin gene (*prd*) tree to examine proteorhodopsin in SAR11. The *psbD* tree is previously published [56]. The *rpoB* tree was updated from a previously published tree [36] with this current paper in mind, but was then used and published in a cyanobacteria focused paper [56]. Here it was visualized with Interactive Tree of Life (iTOL) [57] (Fig. 2). The proteorhodopsin tree (Fig. S1) was started with sequences from a previously published tree [27] and then updated here. The SAR11 DTT-T “proteorhodopsin relative” sequence previously determined not to absorb light [27] was not considered a proteorhodopsin in our analysis. To update these trees, reference amino acid sequences were BLASTed [58] (e-value = 10^−60^) (BLAST v2.6.0+) against assembled protein databases from each location of interest if available. Results of the BLAST search were added to references from previous trees, additional reference sequences from NCBI, and single-cells [59]. Reference sequences were aligned using MUSCLE v3.8.31 [60]. A maximum likelihood tree was made from the alignment using RaxML-ng v0.7.0 [61]. Bootstrap analysis (*n* = 100) was conducted using the transfer bootstrap expectation (tbe) method [62]. Groups within the phylogenetic tree were then labeled based on established subclades of SAR11 [11, 15]. In particular, SAR11 cultures, single cells and MAGs from a previously published SAR11 phylogenetic tree based on core genes [11] were incorporated into our trees and used to identify ecotypes. Additionally, single cells and MAGs from ODZs were incorporated to identify the ODZ-specific ecotypes [15, 32]. Because we incorporated assembled proteins from our stations of interest into the tree, there were some clusters of sequences that did not pertain to known ecotypes. We used the tree topology to name these clusters. For the *rpoB* tree, these new clusters appeared to be linked to the 1b group; additionally, the IIb subclade was broken down further based on tree topology.

For placement of metagenomic reads onto these phylogenetic trees, we followed the same protocol we have used previously [46]. For each gene of interest, representative sequences from each phylogenetically distinct group on the tree were used in a local BLAST (tblastn) search very broadly (e-value = 10^−5^) against databases from each locations’ metagenomic samples. The extracted short reads were then converted to amino acid and aligned to the reference tree using PaPaRa Parsimony-based Phylogeny-Aware Read Alignment program 2.0 [63]. Nonoverlapping paired end reads were then combined into one aligned sequence and placed on the tree by EPA-ng v0.3.5 with filter-max as 1 [64]. Reads placed had a pendant length indicating the similarity between a query read and the location it places on the tree. The reads that placed with a pendant length greater than 2 were removed. Less than 1% of reads were removed. The remaining reads were enumerated for each phylogenetic group using the “assign” subcommand of Gappa v.0.4.0 and a taxonomy file listing the taxonomy of the tree reference sequences [65]. With this method we could separate SAR11 ecotypes and subecotypes (*rpoB*), enumerate both eukaryotic algae and cyanobacteria (*psbD*), and examine the proportion of SAR11 with proteorhodopsin. *psbD* data from GA03 Stations 10, 14, 16, 18, 20, all GA02 stations, and all three HOT stations were previously published [56].

In order to compare across samples and geographic locations read counts were normalized using a previously described method [66]. Normalization factors for each sample were determined by dividing the number of good quality reads in the 100 m ETNP sample by each individual sample. The read counts were multiplied by the sample normalization factor then divided by the length of the gene, and then multiplied by 100 in order to make visualization easier. Phylotype specific depth profiles were created to assess the distribution of each taxonomic group throughout the water column and across geographic locations. Reads were all the same length (150 bp) which made it unnecessary to normalize based on length.

Beta diversity of SAR11 community composition was assessed with a redundancy analysis (RDA) using the vegan package in R V4.4.1 where the environmental variables included depth, nitrate, phosphate, temperature, oxygen, quantity of eukaryotic algae from *psbD*, and the percentage of *psbD* reads that belong to picocyanobacteria. Points on the RDA plot were separated based on the depth regime and whether the sample was in the ODZ. The depths included the euphotic zone, deep euphotic zone, mesopelagic and bathypelagic.

Simpson's Index of Diversity (1-D) [67] was calculated for SAR11 ecotypes to obtain alpha diversity using a presence cutoff of 1 normalized read to reduce noise. The slope of the depth profiles was calculated by dividing the change in ecotype by the change in depth to determine where the changes in diversity had maxima and minima.

Pearson correlations between SAR11 ecotypes and key environmental variables were calculated using the cor function in R and compiled into a correlation matrix (Table S2 DOI 10.6084/m9.figshare.30117898).

## Results and Discussion

**Figure 3:**
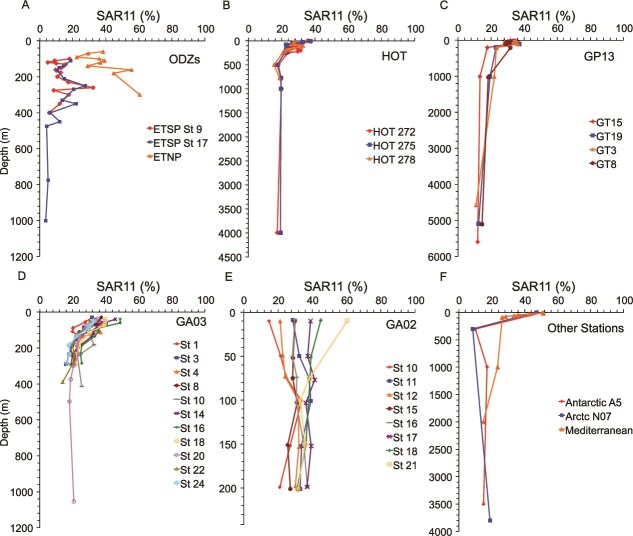
Depth profiles of the percent of the microbial community i.e. SAR11. Profiles are split by ocean region. (A) Depth profiles from the ETNP and ETSP oxygen deficient zones (ODZs). (B) Profiles from the Hawaii Ocean Time series (HOT). (C) Profiles from the Geotraces GP13 cruise in the South Pacific. (D) Profiles from the Geotraces GA03 cruise across the North Atlantic. (E) Profiles from the Geotraces GA02 cruise in the western North Atlantic. (D) Profiles from the polar regions and the Mediterranean Sea.

SAR11 is a broad clade with high diversity [69, 70]. FISH has been used to estimate that SAR11 constitutes ~50% of the community in the surface and 25% in the mesopelagic, based on the Northeast Pacific waters [71]. We analyzed the percent community at a number of depths across four of the world's oceans and many different environmental regimes (Fig. 3). Consistent with previous FISH data [71], we found general euphotic ranges for SAR11 between 30 and 50% of the prokaryotic community, which decreased down to 10%–25% at depth (Fig. 3). However there was no statistical correlation with depth and percent of the microbial community that was SAR11 (*r* = −0.02 *P* = .729). SAR11 was anywhere between 3%–60% of the community but ranges were generally between 20 and 40% (Fig. 3). The smallest variation with depth per station was found in the North Atlantic and largest in the Polar regions (Figs 3D–F). SAR11 in ODZs had both the highest and lowest proportion of the microbial community with only 3% at 1000 m in the ETSP station 17 and 60% at 300 m in the ETNP (Fig. 3A). Our results showed only small variation in the percent community of SAR11 with primary producers (SAR11% community and eukaryotic *psbD r* = 0.00 *P* = .967, picocyanobacterial *psbD r* = 0.14 *P* = .023) and nutrients (*P* = .12 for nitrate, *P* = .6 for phosphate). Previous work which used metagenomic read mapping could not reproduce published FISH results [32], likely due to the lack of good sequenced genomes for deep SAR11 ecotypes and some surface clades. This general agreement between our results and FISH results gives us confidence in our results.

### Environmental variables and SAR11 ecotypes

We constructed a phylogenetic tree of SAR11 using single copy core gene *rpoB* sequences, including assembled proteins from the stations of interest (Fig. 2). Subecotype designations were taken from previously published work [53]. However some assembled proteins from our stations of interest formed clusters not previously known (Fig. 2). The subecotypes designated in this paper from phylogenetic tree topology utilize the letters x, y, and z (Fig. 2A). In particular, we added several subclades to ecotype 1b and split clade IIb into a subclade found primarily in ODZs (IIb.y) and a general deep water subclade (IIb.x), where IIb.x composes most of the group typically referred to as IIb.

**Figure 4:**
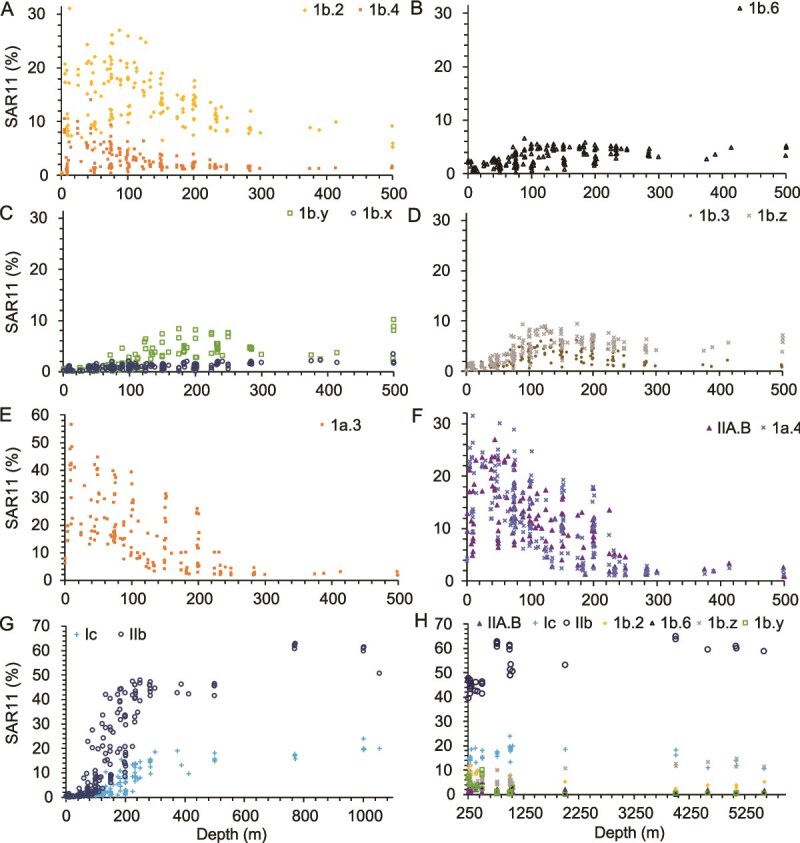
An examination of the effect of depth on different SAR11 ecotypes compiled across all stations. Each graph focuses on a different set of ecotypes: (A) 1b.2 and 1b.4, (B) 1b.6, (C) 1b.y and 1b.x, (D) 1b.3 and 1b.z, (E) 1a.3, (F) IIA.B and 1a.4, (G) Ic and IIb, (H) IIA.B, Ic, 1b.2, 1b.6, 1b.z and 1b.y. Panel H goes from 250 m to 5250 m to highlight the bathypelagic.

When data between cruises were combined, we observed variations between depth profiles of individual ecotypes. SAR11 ecotypes 1a.3, IIA.B, 1a.4, 1b.2, and 1b.4 are most abundant in surface waters (Fig. 4A, C). However, SAR11 ecotypes 1b.6, 1b.3, 1b.y, 1b.z and 1b.x are abundant in the deep euphotic zone and mesopelagic, but not the upper euphotic zone (Figs 4B–E and H). The presence of 1b.2 and 1b.z continues into the deep bathypelagic (Fig. 4C, H). Ecotypes 1c and IIb.x are most abundant in the mesopelagic and bathypelagic (Fig. 4G and H). The ecotypes that are most abundant in surface waters have all been previously identified, but the ecotypes determined here from assembled proteins from the environment are all abundant in the mesopelagic.

**Figure 5:**
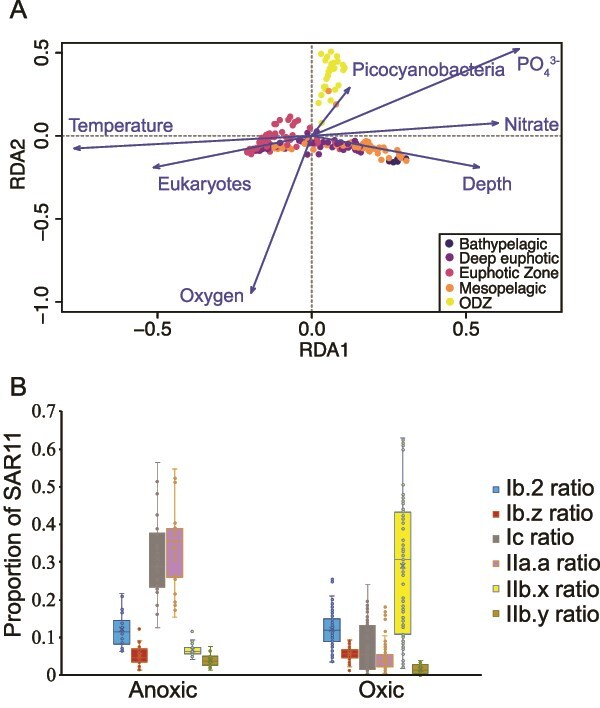
(A) RDA analysis of SAR11 ecotype proportion of SAR11 community compared to environmental factors as well as the abundance of eukaryotic algae and picocyanobacteria as determined by psbD, with the color of each sample reflecting its depth in the water column. PO4 indicates phosphate. (B) Box and whisker plot comparing key SAR11 ecotypes in oxic and anoxic conditions.

To assess the factors that most strongly affected the SAR11 community composition an RDA was run to test the ecotype variation against various environmental factors including temperature, salinity, oxygen, phosphate, nitrate, picocyanobacteria in proportion of the microbial community, eukaryotic algae in proportion of *psbD*, and depth (Fig. 5). These environmental factors constrained 0.59 of the variance in the SAR11 ecotype data. The four most significant factors are oxygen (RDA2 0.95), depth (RDA1 0.68), phosphate (RDA1 0.78), and temperature (RDA1–0.88).

One of the most apparent lobes of points on the RDA aligns with depth (Fig. 5). Depth is constrained in the RDA by both RDA1 (0.608) and RDA2 (0.236). The SAR11 ecotypes that most strongly correlate with depth are IIb.x (*r* = 0.54 *P* = 2.2E-16) and Ib.z (*r* = 0.49 *P* = 2.2E-16) and Ib.2 (*r* = −0.41 *P* = 1.39E-11), which are all ubiquitous at mesopelagic and bathypelagic depths. One might think that more of the surface oriented clades particularly the Ia clades would be more strongly negatively correlated with depth (*r* = 0.02, −0.29, −0.31) but unlike the Ib clades and IIb.x which is, the surface clades are more likely to be sensitive to other external environmental conditions such as temperature than the deep clades. Nutrients and picocyanobacteria abundance are important factors of the RDA (phosphate RDA1 0.78, nitrate RDA1 0.91, picocyanobacteria in proportion of the microbial community RDA1 0.74) and these variables explain more of the surface clade variability.

**Figure 6:**
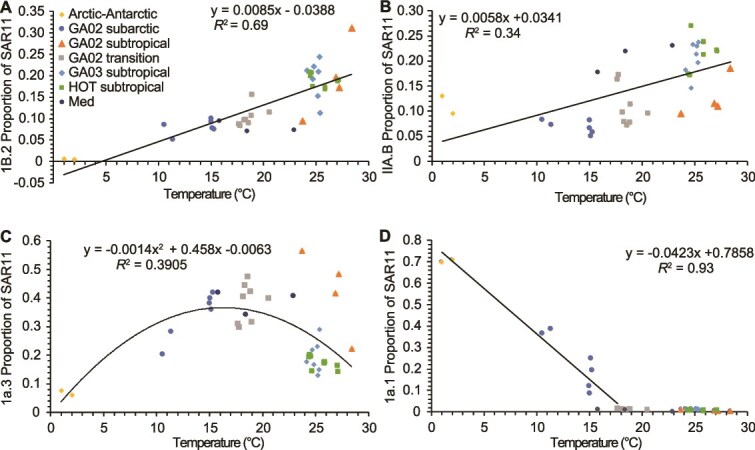
An examination of SAR11 ecotypes in the top 50 m of the water column across our dataset compared to temperature. Each graph focuses on the proportion of SAR11 that was one ecotype (A) 1b.2, (B) IIA.b, (C) 1a.3, and (D) 1a.1. Symbols represent the region where samples were obtained. Data from GP13 in the South Pacific were not included in this analysis, but can be seen in Fig. S40.

Because temperature changes both with latitude and with depth, we wanted to further examine which ecotypes changed with temperature in surface waters (Fig. 6). Specific ecotypes (Ib.2, IIA.B, Ia.3, and Ia.1) abundant in the top 50 m were individually compared to temperature (Fig. 6). SAR11 ecotype Ia.1 is a well-known cold water ecotype that has been found in the polar waters and the more northern waters of the Pacific and Atlantic [14]. Here we see that ecotype Ia.1 was not present in appreciable amounts until the water temperature was <15°C but it then linearly increased until the coldest sample (1°C) with an *R*^2^ = 0.93 (Fig. 6). The proportions of SAR11 that were Ib.2 and IIA.B both consistently increased with increased temperature up to ~20°C with the *R*^2^ values equalling 0.69 and 0.34 respectively. The proportion of SAR11 that was Ia.3 had a unique distribution with temperature compared to the others where it had a peak at 17–18°C and was lower at hotter and colder temperatures (Fig. 6). This type of relationship is more like what is seen during temperature growth curves in the lab [72], and we expect that most surface ecotypes would have similar profiles if their full temperature range was sampled. Data from GP13 was excluded from these analyses because the 1a.3 proportion was unusually low and the 1b.2 proportion was unusually high (Fig. S40). We believe that these differences are due to the >100 m mixed layer at the GP13 stations, while the rest of the dataset had shallower mixed layers [46, 51, 56]. Generally 1a.3 has a steep decline with depth and 1b.2 may have a maximum at 100 m (Fig. 4). The proportions of 1a.3 and 1b.2 are uniform throughout the mixed layer at GP13 (Figs S32–S38) and values for 1a.3 and 1b.2 are more similar to those normal to 100 m at other stations than their respective surface samples (Fig. S40). This may be due to the typically surface oriented ecotypes being exposed to deeper depths and thus lower light levels than is optimal.

The ODZs had a distinct community of SAR11 and the ODZ samples drive the inverse relationship with oxygen (Fig. 5). Similar to previous results [15], the subclades Ic and IIa.A were found to be the dominant subclades in the ETNP and ETSP ODZs respectively, with the other clade being the second most abundant in each case (Fig. 5B, Table S1). IIb.y, a clade identified here by environmental sequences, was also a clade that was found primarily in ODZs, although it was much less prevalent than Ic or IIa.A (Figs S18–S20, S29 and Table S1). Although previous work has shown that Ic is present in ODZs [15], it is generally considered to be the dominant deep water clade [28, 44, 71]. Our data indicated that Ic was present in the mesopelagic but was most prominent in the ODZs and must be well adapted to anoxic and low oxygen conditions (Fig. 5B, depth *r* = 0.19, *P* = .003, oxygen *r* = −0.8 *P* = 2.2E-16, Table S2). Though IIb.y appears to be primarily in the ODZ (oxygen *r* = −0.64 *P* = 2.2E-16), IIb.x was actually generally more abundant than IIb.y in the ODZ (Fig. 5A and B, S18 and S19) and IIb.y was present outside of the ODZs. SAR11 ecotype diversity was elevated in the oxyclines at the edges of the ODZs, where aerobic and anaerobic ecotypes overlapped (Figs S18–S20, S29).

### Group II and deep water clades

**Figure 7:**
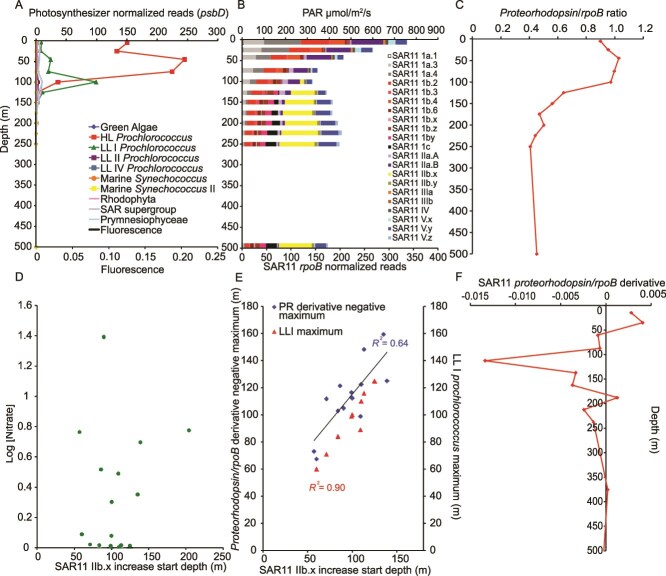
Major depth profile gradients in the HOT 275 (August 2015) dataset. (A) psbD normalized reads of the major primary producers with the chlorophyll fluorescence profile, (B) the rpoB normalized reads of all of the SAR11 ecotypes with the PAR profile overlaid in black, (C) the ratio of SAR11 proteorhodopsin normalized reads to the total SAR11 rpoB normalized reads, indicating the proportion of SAR11 containing proteorhodopsin, (D) the log of the nitrate concentrations versus the depth at which the SAR11 IIb.x clade begins to increase of all stations where the data was available, (E) correlations between the negative maxima of the derivative of the proteorhodopsin to rpoB ratios for each station and the LLI Prochlorococcus maximum for each station both compared against the depth where SAR11 IIb.x clade started to increase for all stations where this information was available, and (F) the derivative of the HOT 275 proteorhodopsin to rpoB normalized read ratio, indicating the depths with the biggest change in the proportion of SAR11 containing proteorhodopsin genes.

Many studies analyze their data at phylogenetic level of clade II [39, 40, 69] or do not include IIb in their analysis [53], but according to our data, Group II is highly diverse and deserves to be analyzed at a finer resolution. In this study, we show that clade II has distinct lineages within it (Fig. 2B) that are important in different locations in the oceans (Figs 4, 5B, 6, and  7). With subclades within clade II being adapted to the mesopelagic and bathypelagic (IIb.x correlation with depth *r* = 0.54, *P* = 2.2E-16, Table S2), high light surface waters (IIa.B correlation with depth *r* = −0.31, *P* = 8.63E-7), anoxic and low oxygen waters (IIa.A correlation with oxygen *r* = −0.78 *P* = 2.20E-16, IIb.y oxygen *r* = −0.64 *P* = 2.2E-16, Table S2), it is likely that these subclades contain different environmentally relevant genetic adaptations, and should not be considered a homogenous group. Due to their abundance and ubiquity, further study of group II as separate subclades would have widespread ecological implications.

Because phylogenetic read placement does not depend on the presence of sequenced genomes, it is an ideal technique to investigate deep water SAR11 ecotypes, several of which are still missing sequenced genomes. Previous studies have asserted Ic as the dominant clade of the mesopelagic and bathypelagic ocean [18, 32, 53, 73]. However, we see much smaller amounts of Ic in the oxic ocean mesopelagic than IIb.x (otherwise known as IIb), which does not have a good sequenced genome. In the oxic ocean at HOT 275, we see that clade Ic makes up 10%–15% of SAR11 below 200 m, and IIb.x increases in proportion from 33% at 200 m to 50% of the SAR11 community at 4000 m (Fig. 7B). We investigated correlations between physical and chemical parameters in order to try and hypothesize the cause for the switch between the more surface clades (Ia, IIa.B, and Ib.2) to the mesopelagic and deeper adapted clades (IIb.x, Ic and Ib.z). When comparing the *rpoB* and *psbD* depth profiles across stations, the depth at which the IIb.x clade begins to increase coincides with the maximum of LLI *Prochlorococcus*, when *Prochlorococcus* was present, with a regression *R*^2^ = 0.90 *P* = 1E-5 (Fig. 7E). Ecotypes within the *Prochlorococcus* genus are ecologically distributed by depth based on light levels [43] with Low Light clades living at depths with reduced light [74]. The maximum in LLI *Prochlorococcus* generally corresponds to the

**Figure 8:**
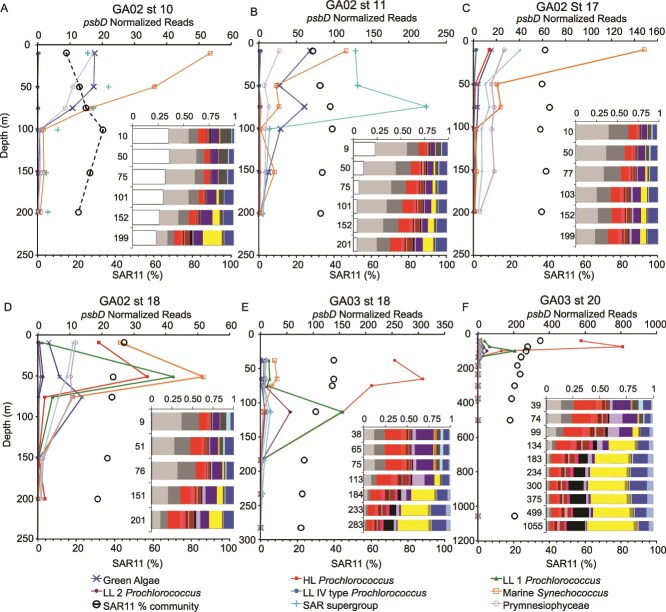
SAR11 abundance compared to primary producers. (A–F) GA02 and GA03 North Atlantic stations with a diverse range of primary producers as indicated by psbD normalized reads and additionally including SAR11% of prokaryotic community (rpoB). Inset: the breakdown of SAR11 ecotype % of SAR11 bar graphs. The legend for SAR11 ecotypes can be seen in Fig. 7. Additional data can be seen in Figs S2–S39.

DCM [46]. DCMs are thought to occur at the balance between light and nutrients, with light being the ultimate control [47]. Indeed, at HOT, the only station where we have PAR (photosynthetic active radiation) data, ecotype IIb.x appeared when PAR was reduced but still present (Fig. 7B). Because the depth of IIb.x begins to increase coinciding with the peak of LLI *Prochlorococcus* (Fig. 7E)*,* we hypothesize that the environmental parameter determining the switch to dominance of the IIb.x clade is light, or a reduction of light levels. We do not think this shift is related to the LLI *Prochlorococcus* itself, as the shift in SAR11 ecotypes occurs even when LLI *Prochlorococcus* is not present in colder waters (Fig. 8).

### Proteorhodopsin

Cultured SAR11, all from surface waters, contain proteorhodopsins [67], which are light-dependent proton pumps [68]. Previous studies focused on the surface ocean, where light is abundant, have examined proteorhodopsin in SAR11 to determine the function of this gene [27, 69]. Proteorhodopsin does not affect the growth of energy replete SAR11 cells, but does aid greatly under energy starved conditions [29]. Reads were placed on a proteorhodopsin tree (Fig. S1) and their phylotypes were evaluated to see if there were changes in SAR11 proteorhodopsin (*prd*) seen at the surface and at depth and the well-known spectral tuning of blue vs green proteorhodopsins [27] were analyzed. The green light proteorhodopsin was present in the surface of the coastal GA03 St 1, Antarctic A5, Arctic N07, and the three northern stations of the GA02 (St 10, 11, 12) (Figs S2, S15–S17) but was not found in the subtropical stations. The proportion of SAR11 with proteorhodopsin did not correlate with nitrate in the top 60 m of the water column (*r* = 0.19, *P* = .18 Table S2) although it did negatively correlate with temperature (*r* = −0.53, *P* = 5.44E-5). These data indicate that in the higher latitudes where the surface water is colder, SAR11 bacteria tend to have proteorhodopsin more than in lower latitudes.

The total abundance of proteorhodopsin in all bacteria has been shown to decrease with depth, and a previous study [27] showed that the proteorhodopsins from below the euphotic zone were distinct from those found in the surface waters. Here we saw a decrease in the proportion of SAR11 containing proteorhodopsin with depth (*r* = −0.34 *P* = 4.26E-8, Fig. 8, Figs S2–S39, Table S1) and the shift in the proteorhodopsin gene abundance coincided with the changes in SAR11 ecotypes with depth. However, proteorhodopsin was present in at least 10% of SAR11 in every sample analyzed. As seen previously [27], the deep proteorhodopsin was phylogenetically distinct from the surface clades in our data (Fig. S1). The blue light proteorhodopsin groups labeled as group 1 and group 4 repeatedly increased with depth and were absent from the surface [27]. Because SAR11 have such streamlined genomes, it seems unlikely that these deep proteorhodopsin groups are related to light harvesting when the clades are adapted to the aphotic zone. In fact a study looking at proteorhodopsin in the North Pacific only found one deep proteorhodopsin that responded to light and that weakly [27]. We took the derivative of the *prd/rpoB* ratio down in the water column to assess where the largest changes in the SAR11 proteorhodopsin profiles occurred. The derivative maximum had a clear correlation with the depth that the deep SAR11 clade IIb.x began to increase and the maximum of LLI *Prochlorococcus* (*R*^2^ = 0.90 *P* = 1E-5) (Fig. 8E). The largest slope (derivative) in the decrease in proportion of SAR11 containing proteorhodopsin with depth coincided well with the increase of IIb.x (*R*^2^ = 0.64 *P* = 9.29E-4) and other deeper water clades (Fig. 8E). This is consistent with the hypothesis that decreasing light with depth is the driving factor in the SAR11 ecotype change. With all of these important changes occurring at the same depth in the lower euphotic zone, it seems that this transitory depth level is an important biogeochemical switch caused by a decrease in light. In contrast, when we investigated correlations between nitrate concentrations and the change between surface and deep ecotypes, we found no correlation (*P* = .65; Fig. 8D). The shift in dominant ecotype of SAR11 happens in the lower euphotic zone rather than in the mesopelagic (Fig. 8A). This means that the shift is occurring not where there is an absence of light but where the light starts to dwindle. SAR11 group V phylogeny is debated and is known to have diverging proteorhodopsin and can have multiple copies [31]. In the SAR11 section of the *prd* tree, group V formed a group within the outgroups and was not included in our analysis (Fig. S1).

### Eukaryotic influence

SAR11 cannot synthesize its own reduced organic S compounds or vitamin B_1_ [7–9]. Previous studies have co-cultured SAR11 with eukaryotic algae to show that eukaryotic algae can provide simple reduced organic S compounds, such as DMSP, taurine, HMP, as well as vitamin B_1_ and volatile organic C to SAR11 bacteria [9, 75–77]. In a similar co-culturing study, *Prochlorococcus* could not provide SAR11 with all needed reduced organic S compounds [78]. Therefore, one might expect SAR11 proportion of community or ecotype composition to correlate with eukaryotic algae. However, although sometimes the proportion of the microbial community that was SAR11 did decrease from the surface to the bathypelagic, this decrease was gradual and profiles did not have a sharp drop off at the bottom of the euphotic zone (Fig. 3). When comparing the spring GA02 transect, dominated by eukaryotes and *Synechococcus,* to the fall GA03 transect dominated by *Prochlorococcus*, profiles of the proportion of SAR11 did not alter despite the large differences in the most abundant primary producers (Fig. 8, Table S2). There was no correlation between Eukaryotic algae as determined by *psbD* and the proportion of SAR11 (*r* = 0.00 *P* = .96, Table S2). There was a slight correlation between Picocyanobacteria *psbD* and the SAR11 proportion of the community (*r* = 0.14 *P* = .02, Table S2) but it was not strong.

Although we did not see strong correlations between Picocyanobacteria or Eukaryotes and the SAR11 proportion of the microbial community, we do have some correlations that align with the primary producers and specific SAR11 ecotypes. Eukaryotic *psbD* correlated most strongly with SAR11 groups 1a.3 (*r* = 0.62 *P* = 2.20E-16 Supplemental table 2) and group 1a.4 (*r* = 0.46, *P* = 1.70E-14 Supplemental table 2) with smaller negative correlations with IIb.x (*r* = −0.35 *P* = 1.08E-8 Supplemental table 2) and IIb.y (*r* = −0.34 *P* = 2.63E-8 Supplemental table 2). Picocyanobacteria *psbD* correlated both more strongly and with more specific SAR11 ecotypes. The ecotypes that most strongly correlated with Picocyanobacteria were Ib.4 (*r* = 0.87 *P* = 2.2E-16) IIaB (*r* = 0.70 *P* = 2.2 E-16) and Ib.2 (*r* = 0.69 *P* = 2.2E-7) but there were a few other SAR11 clades that had significant correlations above 0.5 (Supplemental table 2). All of the strongest positive correlations between primary producers and the SAR11 clades align with clades that are significantly correlated with shallow depths (Supplemental Table 2; Fig. 4). Because SAR11 clades occupy specific depths and temperature regimes, it is difficult to distinguish between co-correlations and actual linkages, especially with surface oriented clades. These clades may rely more heavily on the reduced S and vitamins provided from the primary producers at the surface while the clades found throughout the water column or at depths below the euphotic zone may be relying on other sources. More studies need to be done to determine other sources of reduced organic S and vitamins in the marine environment.

## Conclusions

Phylogenetic read placement of single copy core genes allows for differentiation of bacterial ecotypes that other metagenomic techniques are not able to resolve when complete genomes are lacking. This paired with the analysis of deep depth profiles reveals that the global distribution of SAR11 and its ecotypes have distinct distributions based on light, depth, temperature, and oxygen. Clades were found to be specific to the meso/bathypelagic, ODZs, polar regions, and surface waters. Within clade II, which is often analyzed as a single entity, we found subclades that occupied very different niches including euphotic surface waters (IIa.B), ODZs (IIa.A, IIb.y) and the deeper meso and bathypelagic (IIb.x). Clade IIb.x, rather than 1c, dominate mesopelagic and bathypelagic waters. Understanding the meso and bathypelagic waters is crucial to understanding the world's oceans and their carbon cycling due to the large volumes of these regions compared to the productive surface waters. The proteorhodopsin/*rpoB* ratio showed that there was a decrease in the proportion of SAR11 carrying the proteorhodopsin gene below the euphotic zone. In fact, the depth of greatest change in proportion of SAR11 with proteorhodopsin was the same depth where clade structure changed from surface to deep ecotypes and was also coincident with the maximum in LLI *Prochlorococcus*, a low light ecotype of picocyanobacteria, leading us to believe that the transition between ecotype regimes is due to light levels (Fig. 8). This transition occurs in the lower euphotic zone, rather than in the true mesopelagic.

Regardless of the dominant primary producer, the SAR11% community and ecotype structure remained fairly consistent in the euphotic zone with consistent surface clades. Therefore, although photosynthesizers, specifically Eukaryotes, are a good source of necessary reduced S and vitamins, additional sources must be available in the environment in oligotrophic and deeper waters. Further work must be done to more clearly understand why individual ecotypes are more suited for certain environments, and how specific metabolic needs are being met in these environments.

## Supplementary Material

Supplemental_table_fig_wraf221

## Data Availability

Metagenomes were downloaded from the Hawaii Ocean Time series (HOT) (Bioproject PRJNA352737), the ETNP ODZ [36] (Bioproject PRJNA350692), two stations from the ETSP ODZ (Bioproject PRJNA704804), four stations from Geotraces GP13 in the South Pacific (Bioproject PRJNA385854), one station in the Arctic and one station in the Amundsen Sea in Southern Ocean near Antarctica (Bioproject number PRJNA588686), 13 stations from the North Atlantic Geotraces GA03 transect and seven stations from the North Atlantic Geotraces GA02 transect (Bioproject PRJNA385854), and one station in the Mediterranean Sea from October 2015 [53] (BioProject PRJNA352798). For Geotraces cruises (GA02, GA03, and GP13), CTD and nutrient data were downloaded from the British Oceanographic Data Centre (https://www.bodc.ac.uk/geotraces/) as part of the GEOTRACES 2021 Intermediate Data Product (IDP2021). SRR numbers and all relevant metadata are included in Supplemental Table 1. Scripts used for making figures can be found at DOI 10.6084/m9.figshare.30117721 (Map Fig. 1) and DOI 10.6084/m9.figshare.30117880 (RDA Fig. 5A). Alignments and FASTA files for all data used to construct trees are available for *rpoB* (DOI 10.6084/m9.figshare.30117886), *psbD* (DOI 10.6084/m9.figshare.30117883) and *prd* (DOI 10.6084/m9.figshare.30117889). The detailed protocol for phylogenetic read placement and all the scripts involved can be found at protocols.io [55]. SAR11 ecotype data from the RNA polymerase gene (*rpoB*) and proteorhodopsin (*prd*) can be found in Supplemental Table 1 (DOI 10.6084/m9.figshare.30117754). Supplemental figures can be found on figshare using DOI 10.6084/m9.figshare.30117904.
