# Supplementary material for: SAR11 ecotypes across ocean basins change with depth due to changes in light and oxygen
Source: ISME J. 2025 Oct 8;19(1):wraf221. doi: 10.1093/ismejo/wraf221 (PMC12598773; doi:10.1093/ismejo/wraf221)
Supplement: Supplemental_table_fig_wraf221 [file supplemental_table_fig_wraf221.zip › Supplemental_table_fig_wraf221/Supplemental_figures_2_40.pdf]

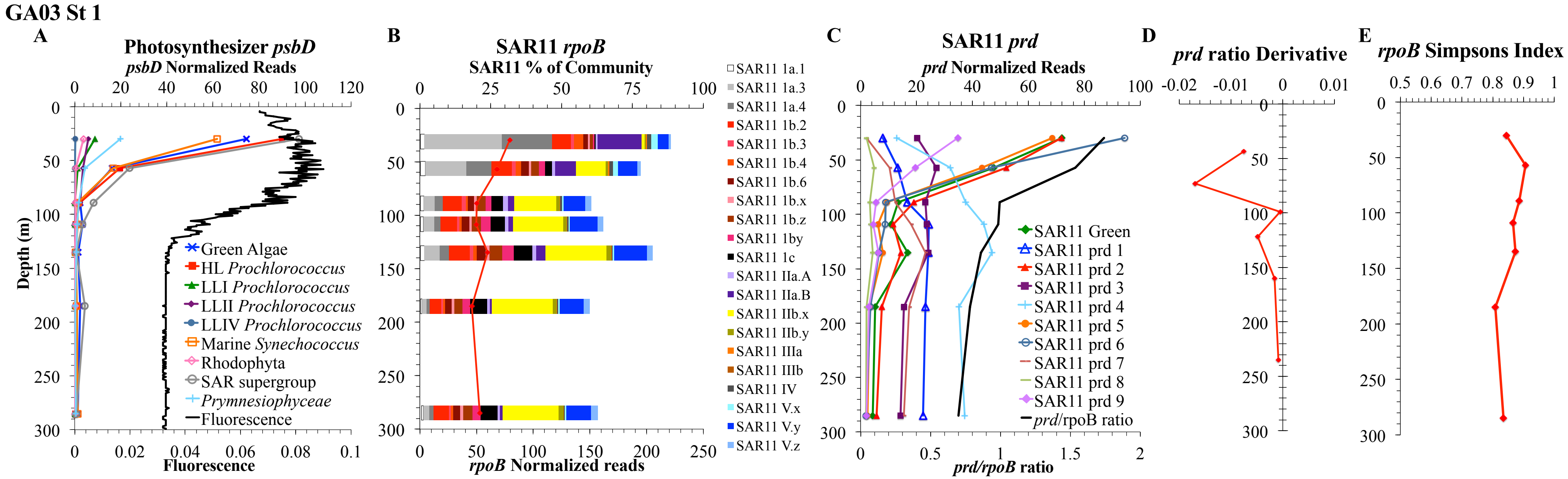

Supplemental Fig 2: Relevant analyses of the Geotraces GA03 St 1 metagenomic depth profile. A) Normalized reads for photosystem II reaction center protein D2 (*psbD)* for the most common photosynthesizers and chlorophyll fluorescence. B) SAR11 RNA polymerase subunit beta (*rpoB*) normalized reads and SAR11 percent of microbial community calculated from *rpoB* normalized reads. C) Proteorhodopsin (*prd*) normalized reads and *prd/rpoB* ratio. D) Derivative of *prd/rpoB* to show where largest changes in the ratio occur in the water column. E) Simpsons Index of Diversity of SAR11 ecotypes based on *rpoB* normalized reads.

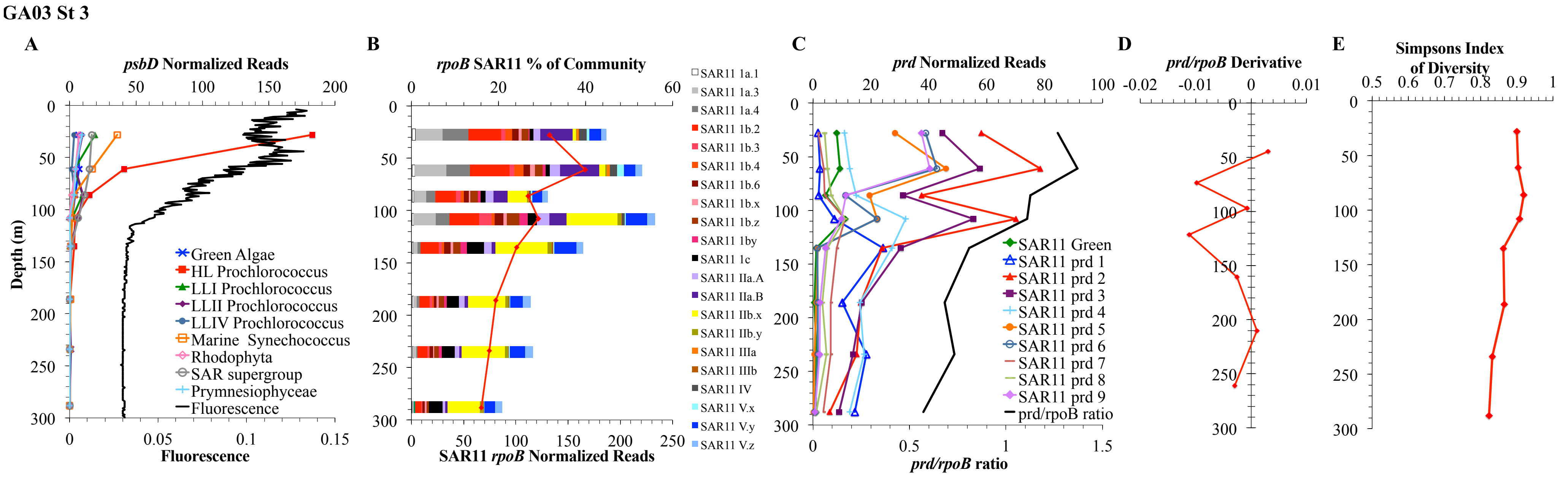

Supplemental Fig 3: Relevant analyses of the Geotraces GA03 St 3 metagenomic depth profile. A) Normalized reads for photosystem II reaction center protein D2 (*psbD)* for the most common photosynthesizers and chlorophyll fluorescence. B) SAR11 RNA polymerase subunit beta (*rpoB*) normalized reads and SAR11 percent of microbial community calculated from *rpoB* normalized reads. C) Proteorhodopsin (*prd*) normalized reads and *prd/rpoB* ratio. D) Derivative of *prd/rpoB* to show where largest changes in the ratio occur in the water column. E) Simpsons Index of Diversity of SAR11 ecotypes based on *rpoB* normalized reads.

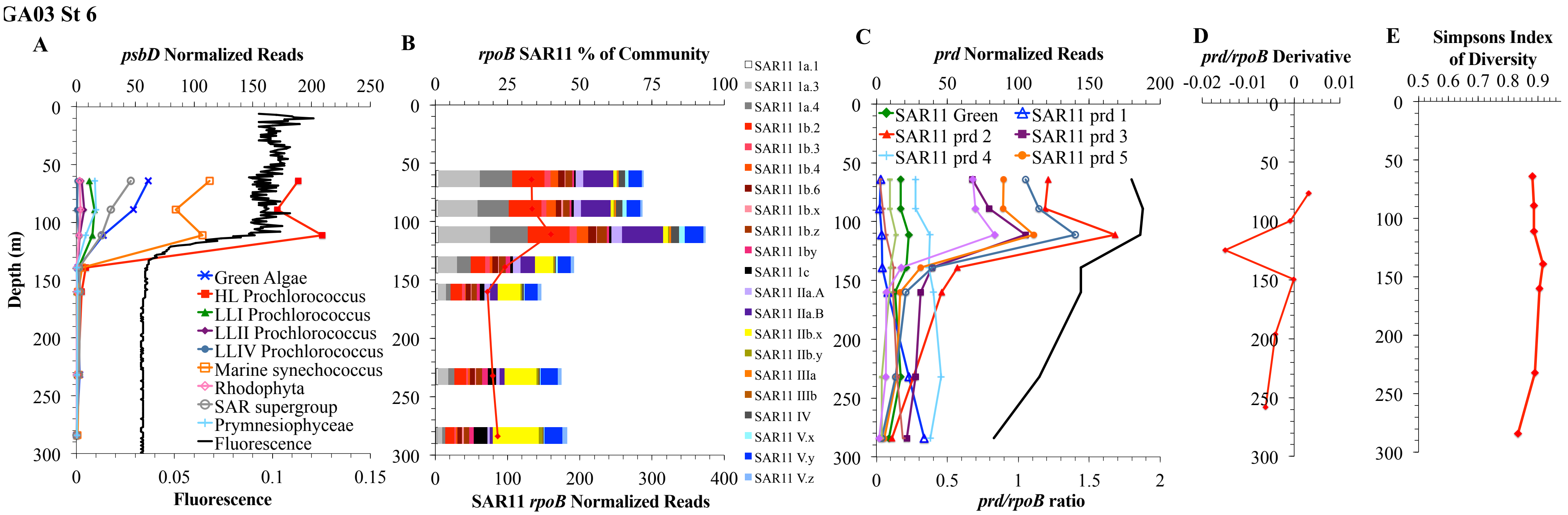

Supplemental Fig 4: Relevant analyses of the Geotraces GA03 St 6 metagenomic depth profile. A) Normalized reads for photosystem II reaction center protein D2 (*psbD)* for the most common photosynthesizers and chlorophyll fluorescence. B) SAR11 RNA polymerase subunit beta (*rpoB*) normalized reads and SAR11 percent of microbial community calculated from *rpoB* normalized reads. C) Proteorhodopsin (*prd*) normalized reads and *prd/rpoB* ratio. D) Derivative of *prd/rpoB* to show where largest changes in the ratio occur in the water column. E) Simpsons Index of Diversity of SAR11 ecotypes based on *rpoB* normalized reads.

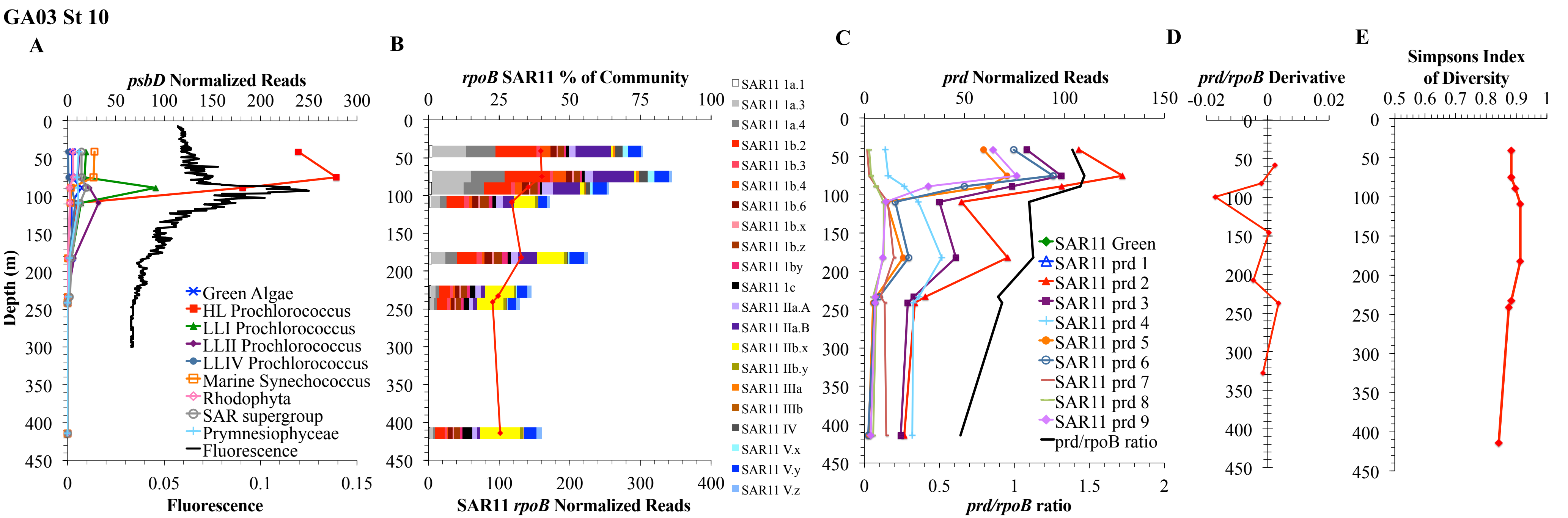

Supplemental Fig 5: Relevant analyses of the Geotraces GA03 St 10 metagenomic depth profile. A) Normalized reads for photosystem II reaction center protein D2 (*psbD)* for the most common photosynthesizers and chlorophyll fluorescence. B) SAR11 RNA polymerase subunit beta (*rpoB*) normalized reads and SAR11 percent of microbial community calculated from *rpoB* normalized reads. C) Proteorhodopsin (*prd*) normalized reads and *prd/rpoB* ratio. D) Derivative of *prd/rpoB* to show where largest changes in the ratio occur in the water column. E) Simpsons Index of Diversity of SAR11 ecotypes based on *rpoB* normalized reads.

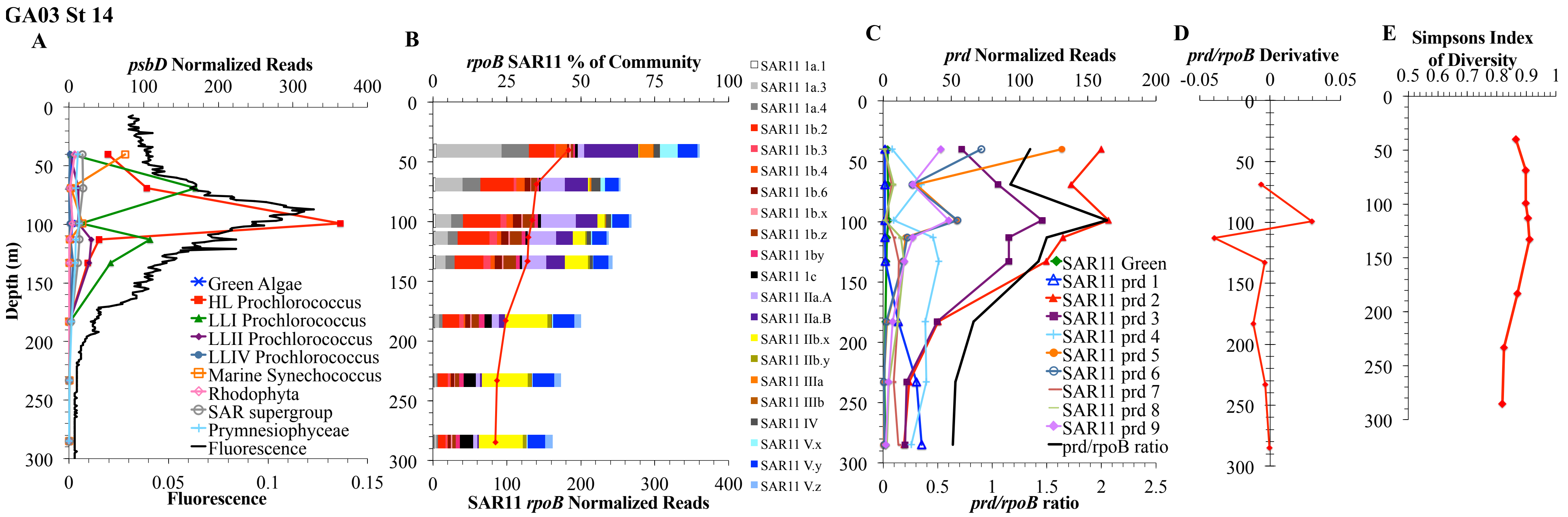

Supplemental Fig 6: Relevant analyses of the Geotraces GA03 St 14 metagenomic depth profile. A) Normalized reads for photosystem II reaction center protein D2 (*psbD)* for the most common photosynthesizers and chlorophyll fluorescence. B) SAR11 RNA polymerase subunit beta (*rpoB*) normalized reads and SAR11 percent of microbial community calculated from *rpoB* normalized reads. C) Proteorhodopsin (*prd*) normalized reads and *prd/rpoB* ratio. D) Derivative of *prd/rpoB* to show where largest changes in the ratio occur in the water column. E) Simpsons Index of Diversity of SAR11 ecotypes based on *rpoB* normalized reads.

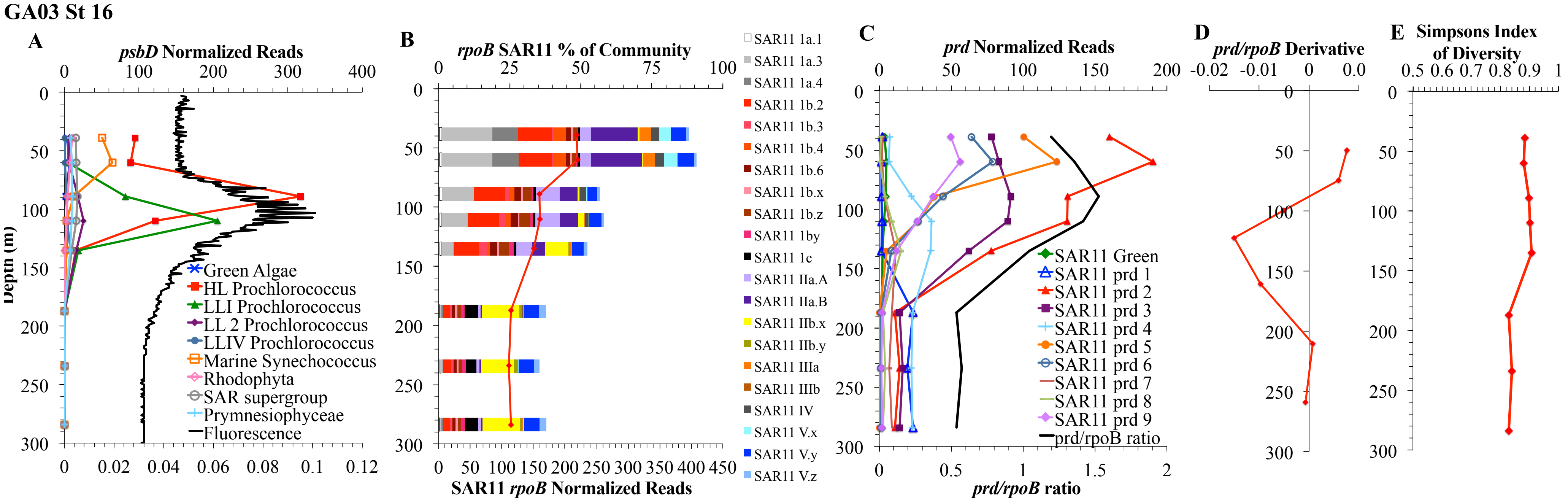

Supplemental Fig 7: Relevant analyses of the Geotraces GA03 St 16 metagenomic depth profile. A) Normalized reads for photosystem II reaction center protein D2 (*psbD)* for the most common photosynthesizers and chlorophyll fluorescence. B) SAR11 RNA polymerase subunit beta (*rpoB*) normalized reads and SAR11 percent of microbial community calculated from *rpoB* normalized reads. C) Proteorhodopsin (*prd*) normalized reads and *prd/rpoB* ratio. D) Derivative of *prd/rpoB* to show where largest changes in the ratio occur in the water column. E) Simpsons Index of Diversity of SAR11 ecotypes based on *rpoB* normalized reads.

GA03 St 18

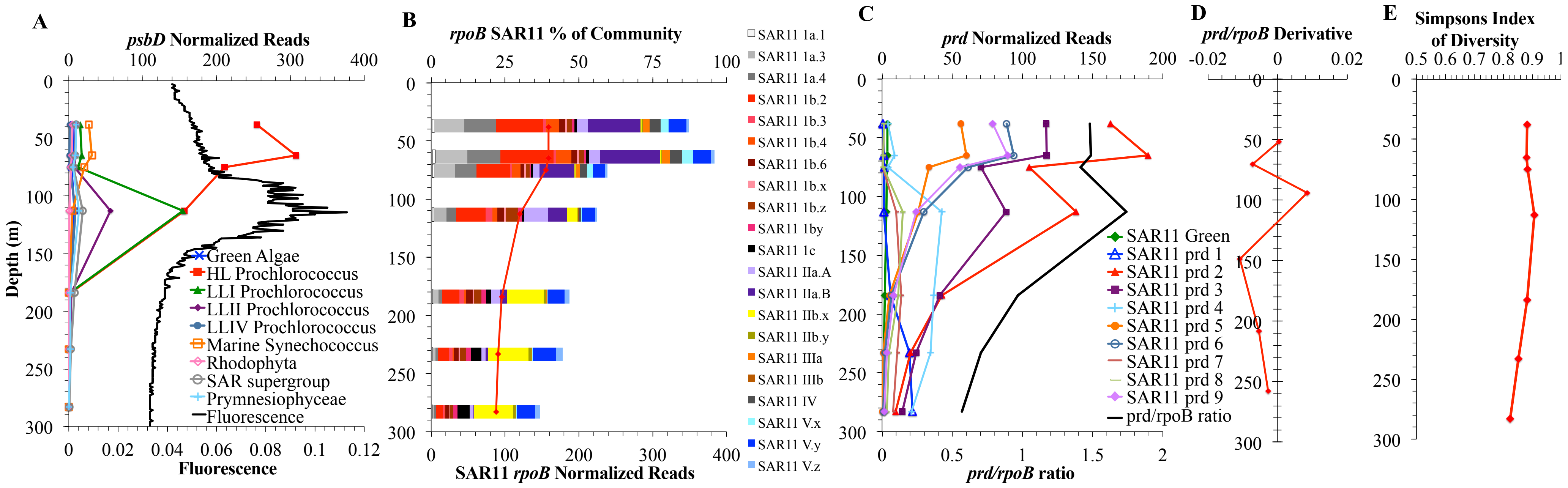

Supplemental Fig 8: Relevant analyses of the Geotraces GA03 St 18 metagenomic depth profile. A) Normalized reads for photosystem II reaction center protein D2 (*psbD)* for the most common photosynthesizers and chlorophyll fluorescence. B) SAR11 RNA polymerase subunit beta (*rpoB*) normalized reads and SAR11 percent of microbial community calculated from *rpoB* normalized reads. C) Proteorhodopsin (*prd*) normalized reads and *prd/rpoB* ratio. D) Derivative of *prd/rpoB* to show where largest changes in the ratio occur in the water column. E) Simpsons Index of Diversity of SAR11 ecotypes based on *rpoB* normalized reads.

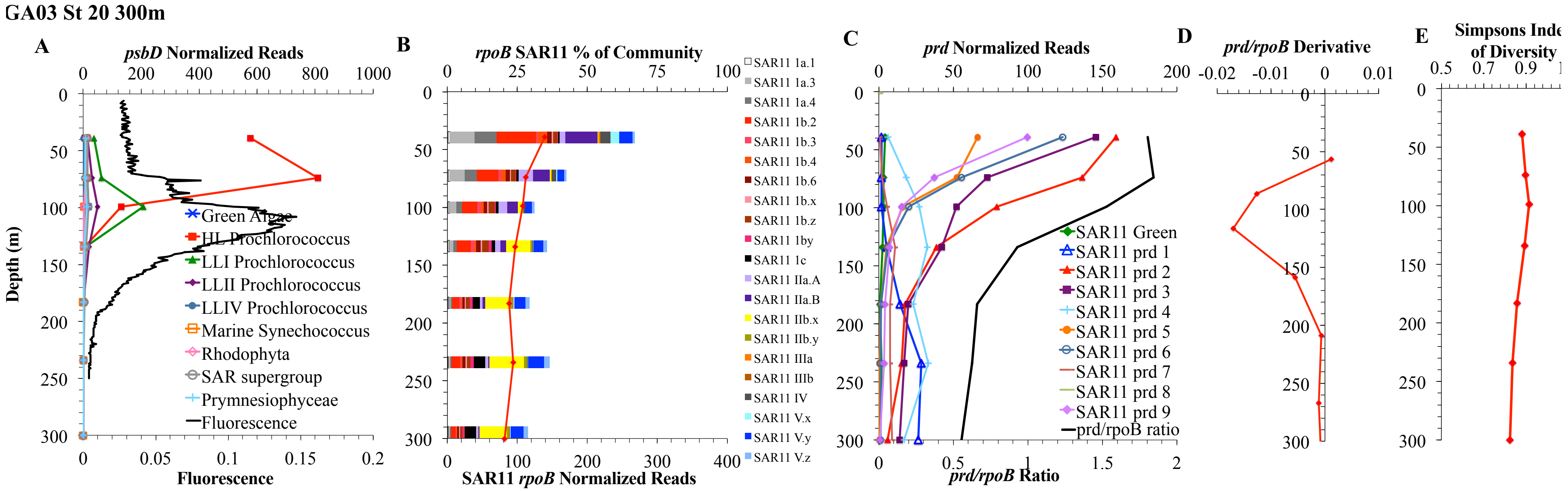

Supplemental Figure 9: Relevant analyses of the GA03 Station 20 metagenomic depth profile showing the top 300 m. A) Normalized reads for photosystem II reaction center protein D2 (*psbD)* for the most common photosynthesizers and chlorophyll fluorescence. B) SAR11 RNA polymerase subunit beta (*rpoB*) normalized reads and SAR11 percent of microbial community calculated from *rpoB* normalized reads. C) Proteorhodopsin (*prd*) normalized reads and *prd/rpoB* ratio. D) Derivative of *prd/rpoB* to show where largest changes in the ratio occur in the water column. E) Simpsons Index of Diversity of SAR11 ecotypes based on *rpoB* normalized reads.

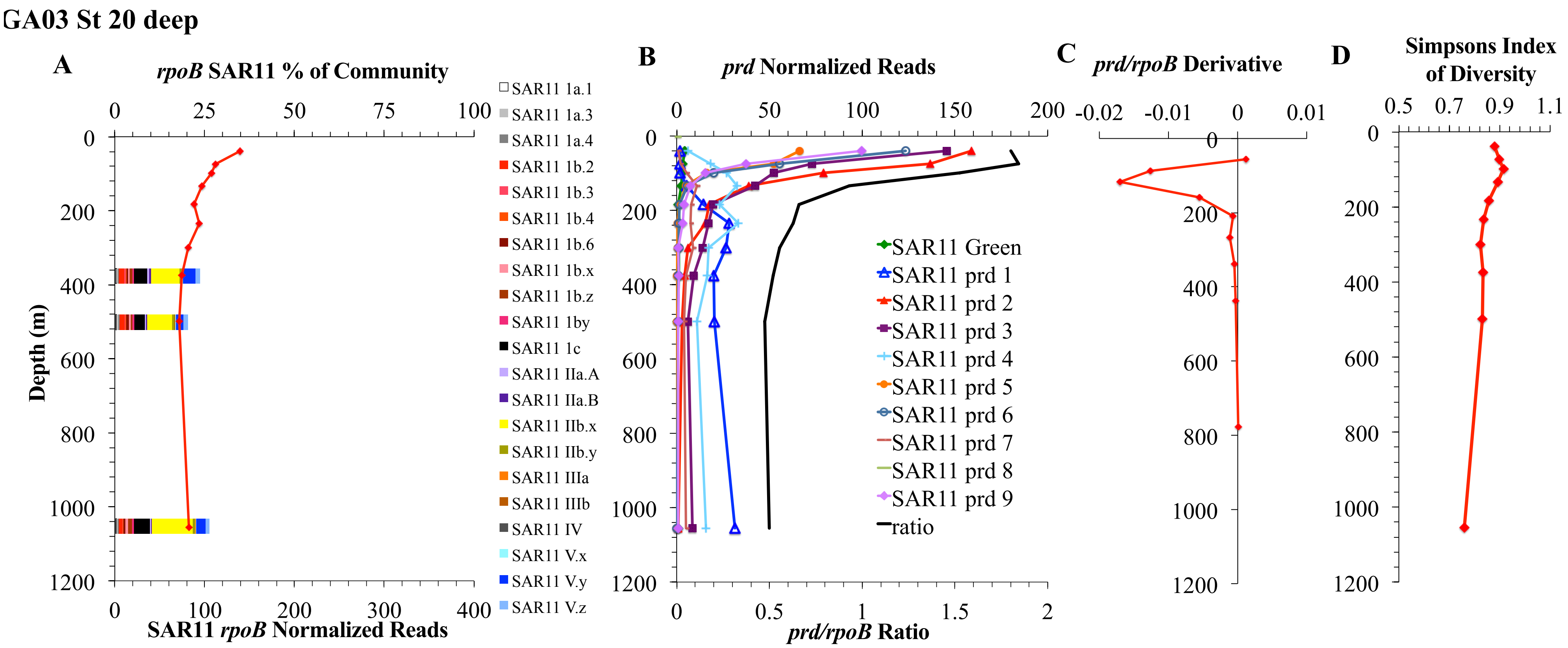

Supplemental Figure 10: Relevant analyses of the GA03 Station 20 metagenomic depth profile showing the entire sampled water column. A) SAR11 RNA polymerase subunit beta (*rpoB*) normalized reads and SAR11 percent of microbial community calculated from *rpoB* normalized reads. B) Proteorhodopsin (*prd*) normalized reads and *prd/rpoB* ratio. C) Derivative of *prd/rpoB* to show where largest changes in the ratio occur in the water column. D) Simpsons Index of Diversity of SAR11 ecotypes based on *rpoB* normalized reads.

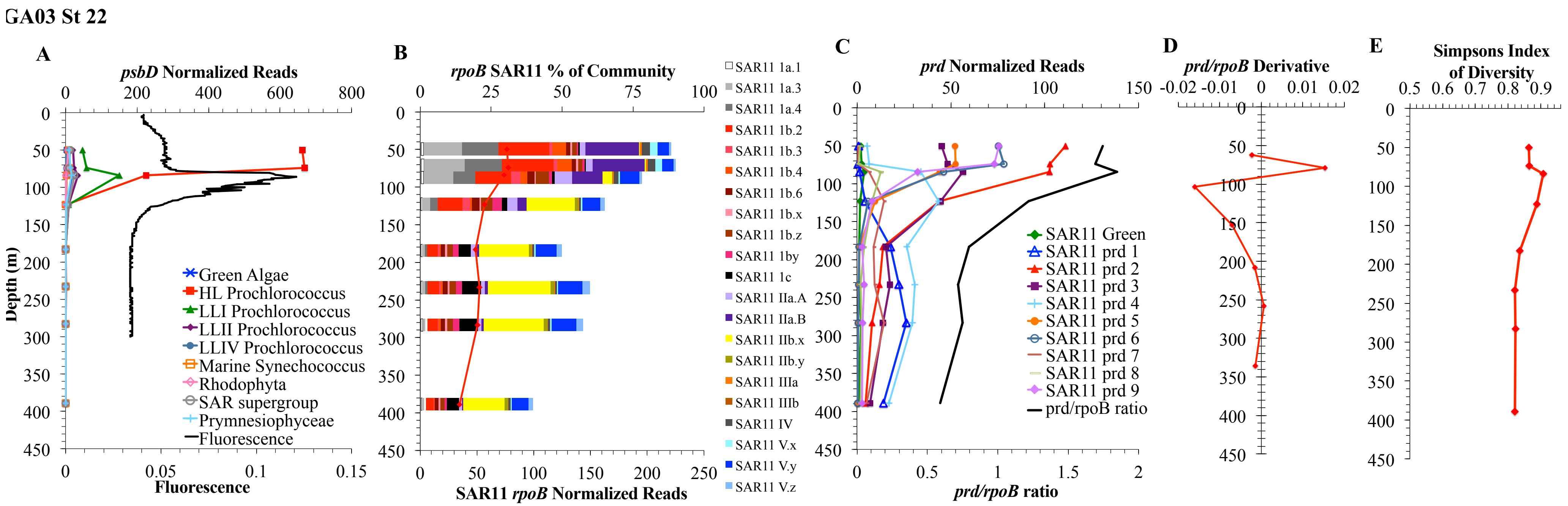

Supplemental Fig 11: Relevant analyses of the Geotraces GA03 St 22 metagenomic depth profile. A) Normalized reads for photosystem II reaction center protein D2 (*psbD)* for the most common photosynthesizers and chlorophyll fluorescence. B) SAR11 RNA polymerase subunit beta (*rpoB*) normalized reads and SAR11 percent of microbial community calculated from *rpoB* normalized reads. C) Proteorhodopsin (*prd*) normalized reads and *prd/rpoB* ratio. D) Derivative of *prd/rpoB* to show where largest changes in the ratio occur in the water column. E) Simpsons Index of Diversity of SAR11 ecotypes based on *rpoB* normalized reads.

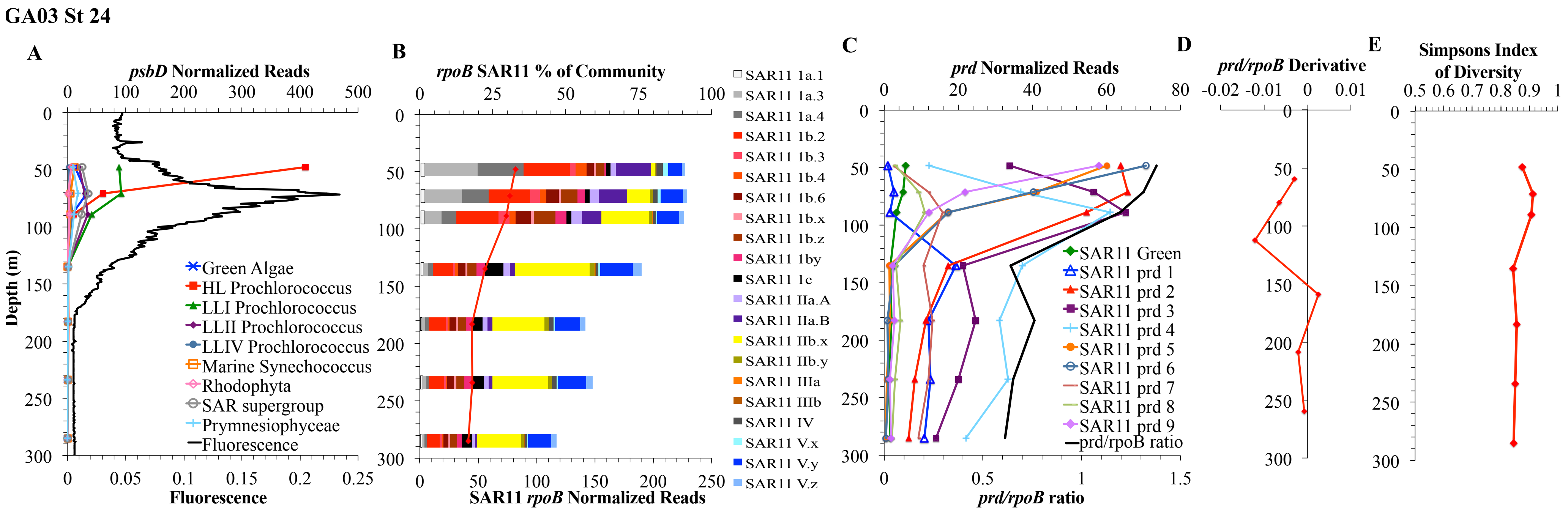

Supplemental Fig 12: Relevant analyses of the Geotraces GA03 St 24 metagenomic depth profile. A) Normalized reads for photosystem II reaction center protein D2 (*psbD)* for the most common photosynthesizers and chlorophyll fluorescence. B) SAR11 RNA polymerase subunit beta (*rpoB*) normalized reads and SAR11 percent of microbial community calculated from *rpoB* normalized reads. C) Proteorhodopsin (*prd*) normalized reads and *prd/rpoB* ratio. D) Derivative of *prd/rpoB* to show where largest changes in the ratio occur in the water column. E) Simpsons Index of Diversity of SAR11 ecotypes based on *rpoB* normalized reads.

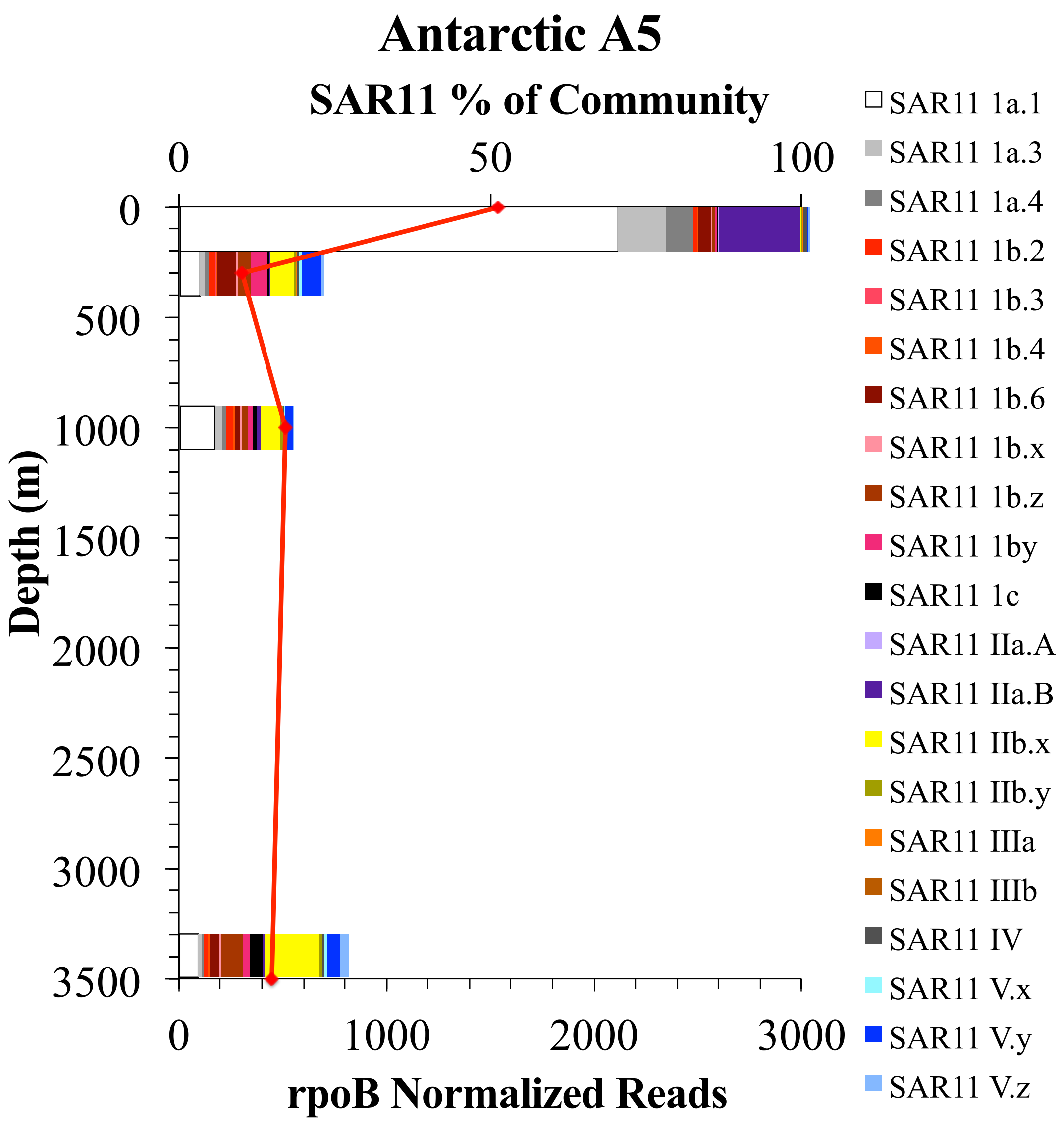

Supplemental Figure 13: *rpoB* normalized reads and % of the microbial community that is made up of SAR11 in the Antarctic station A5.

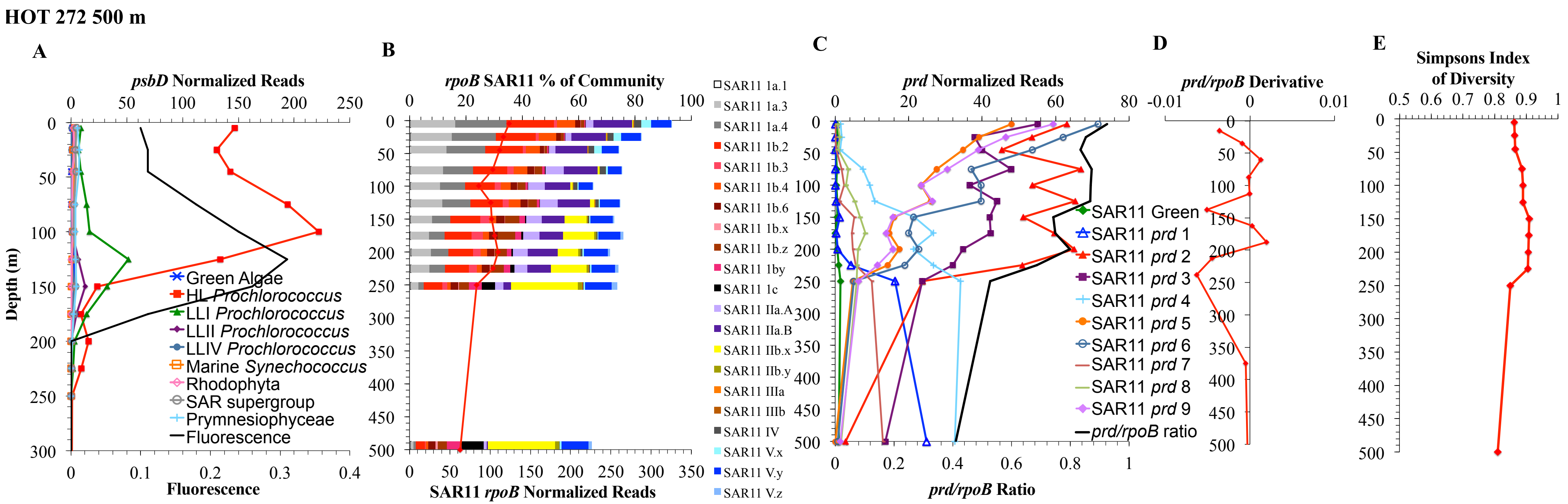

Supplemental Figure 14: Relevant analyses of the HOT 272 (Hawaii Ocean Time Series May 2015) metagenomic depth profile showing the top 500 m. A) Normalized reads for photosystem II reaction center protein D2 (*psbD)* for the most common photosynthesizers and chlorophyll fluorescence. B) SAR11 RNA polymerase subunit beta (*rpoB*) normalized reads and SAR11 percent of microbial community calculated from *rpoB* normalized reads. C) Proteorhodopsin (*prd*) normalized reads and *prd/rpoB* ratio. D) Derivative of *prd/rpoB* to show where largest changes in the ratio occur in the water column. E) Simpsons Index of Diversity of SAR11 ecotypes based on *rpoB* normalized reads.

HOT 272 Deep

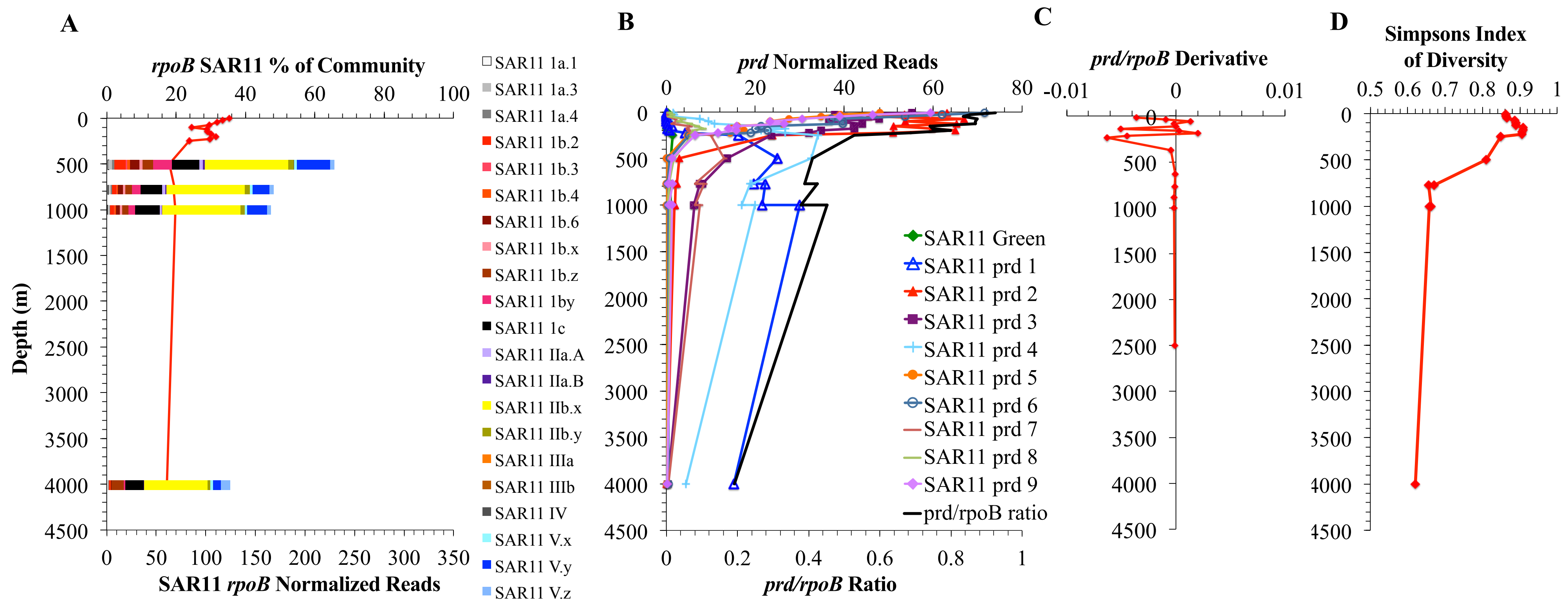

Supplemental Figure 15: Relevant analyses of the HOT 272 (Hawaii Ocean Time Series May 2015) metagenomic depth profile showing the entire sampled water column. A) SAR11 RNA polymerase subunit beta (*rpoB*) normalized reads and SAR11 percent of microbial community calculated from *rpoB* normalized reads. B) Proteorhodopsin (*prd*) normalized reads and *prd/rpoB* ratio. C) Derivative of *prd/rpoB* to show where largest changes in the ratio occur in the water column. D) Simpsons Index of Diversity of SAR11 ecotypes based on *rpoB* normalized reads.

HOT 278 500 m

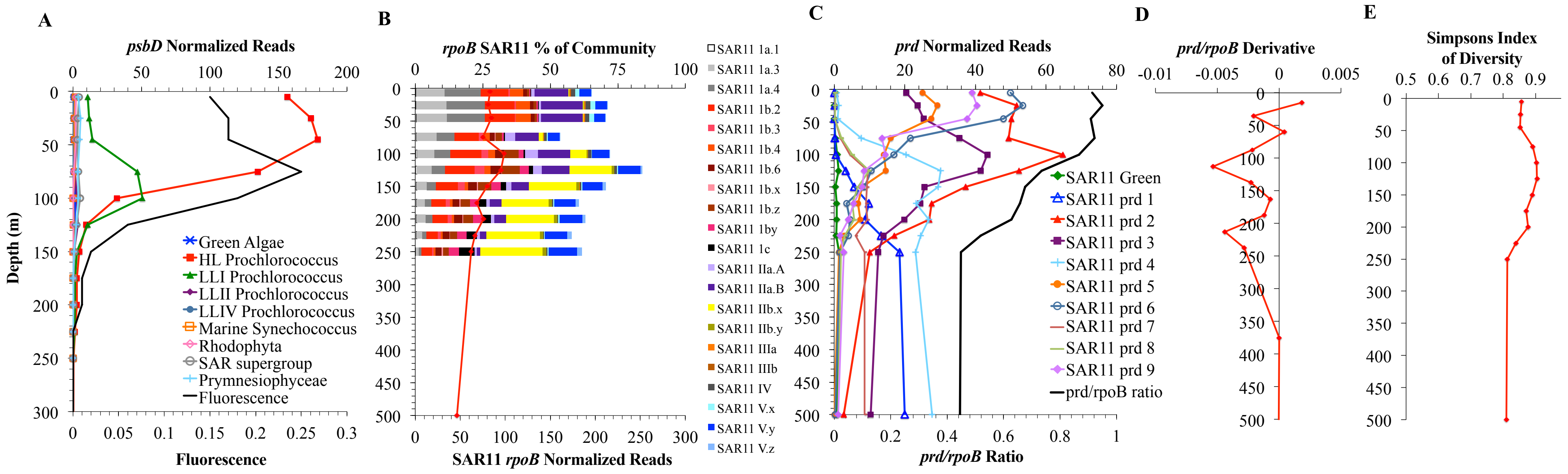

Supplemental Figure 16: Relevant analyses of the HOT 278 (Hawaii Ocean Time Series May 2015) metagenomic depth profile showing the top 500 m. A) Normalized reads for photosystem II reaction center protein D2 (*psbD)* for the most common photosynthesizers and chlorophyll fluorescence. B) SAR11 RNA polymerase subunit beta (*rpoB*) normalized reads and SAR11 percent of microbial community calculated from *rpoB* normalized reads. C) Proteorhodopsin (*prd*) normalized reads and *prd/rpoB* ratio. D) Derivative of *prd/rpoB* to show where largest changes in the ratio occur in the water column. E) Simpsons Index of Diversity of SAR11 ecotypes based on *rpoB* normalized reads.

HOT 278 Deep

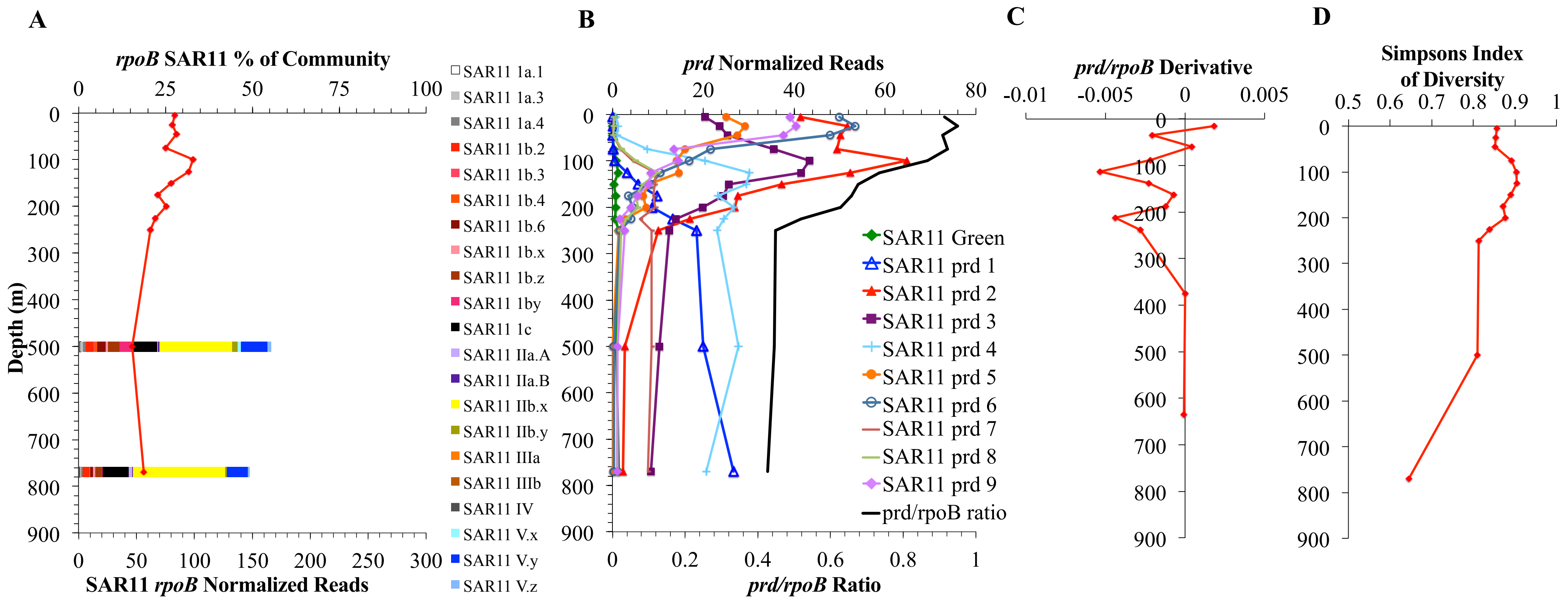

Supplemental Figure 17: Relevant analyses of the HOT 278 (Hawaii Ocean Time Series May 2015) metagenomic depth profile showing the entire sampled water column. A) SAR11 RNA polymerase subunit beta (*rpoB*) normalized reads and SAR11 percent of microbial community calculated from *rpoB* normalized reads. B) Proteorhodopsin (*prd*) normalized reads and *prd/rpoB* ratio. C) Derivative of *prd/rpoB* to show where largest changes in the ratio occur in the water column. D) Simpsons Index of Diversity of SAR11 ecotypes based on *rpoB* normalized reads.

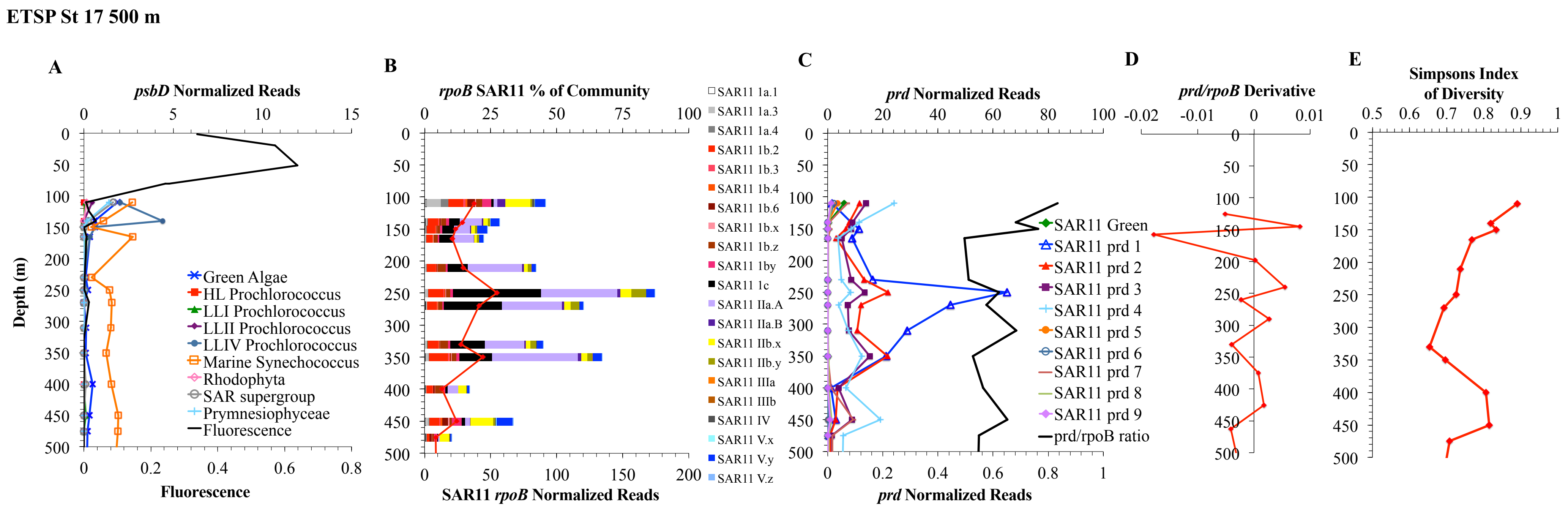

Supplemental Figure 18: Relevant analyses of the ETSP St 17 metagenomic depth profile showing the top 500 m. A) Normalized reads for photosystem II reaction center protein D2 (*psbD)* for the most common photosynthesizers and chlorophyll fluorescence. B) SAR11 RNA polymerase subunit beta (*rpoB*) normalized reads and SAR11 percent of microbial community calculated from *rpoB* normalized reads. C) Proteorhodopsin (*prd*) normalized reads and *prd/rpoB* ratio. D) Derivative of *prd/rpoB* to show where largest changes in the ratio occur in the water column. E) Simpsons Index of Diversity of SAR11 ecotypes based on *rpoB* normalized reads.

ETSP St 17 Deep

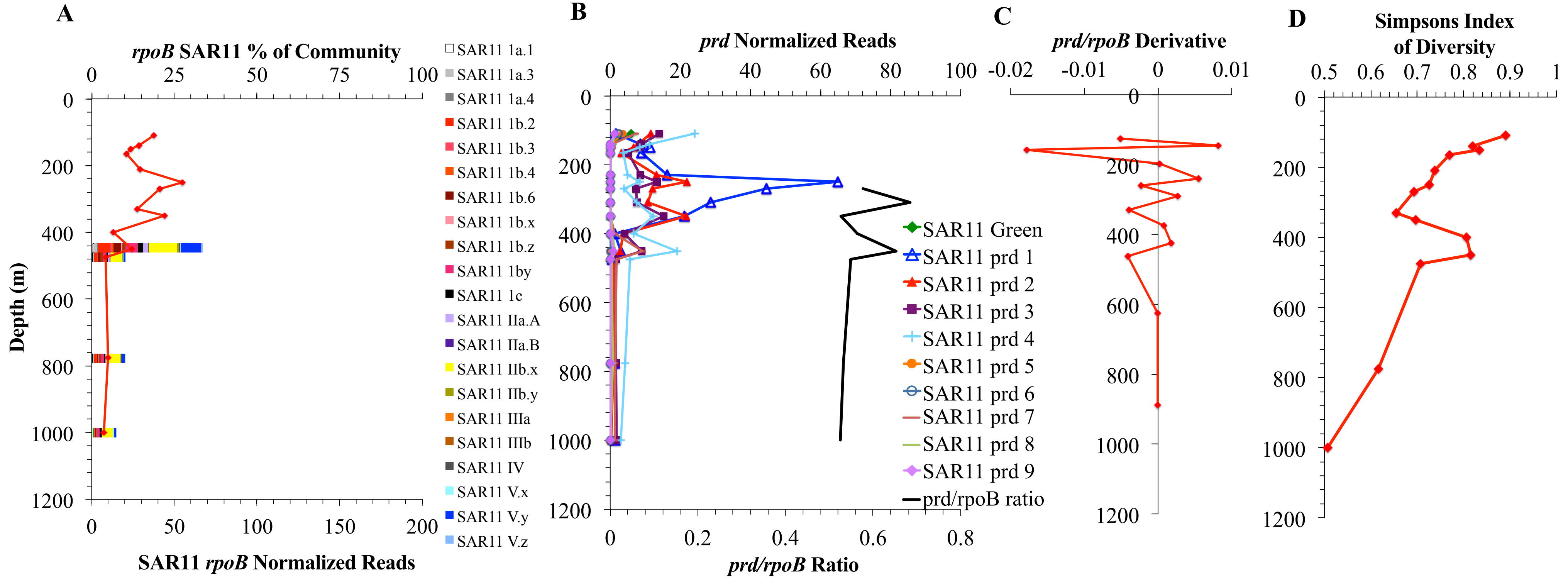

Supplemental Figure 19: Relevant analyses of the ETSP St 17 metagenomic depth profile showing the entire sampled water column. A) SAR11 RNA polymerase subunit beta (*rpoB*) normalized reads and SAR11 percent of microbial community calculated from *rpoB* normalized reads. B) Proteorhodopsin (*prd*) normalized reads and *prd/rpoB* ratio. C) Derivative of *prd/rpoB* to show where largest changes in the ratio occur in the water column. D) Simpsons Index of Diversity of SAR11 ecotypes based on *rpoB* normalized reads.

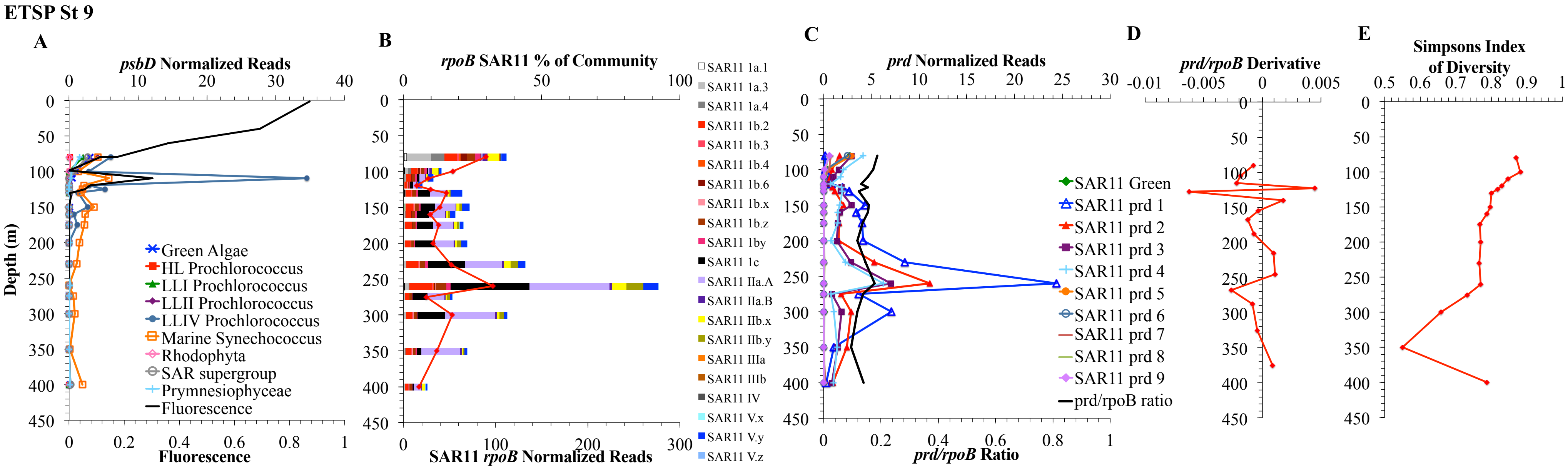

Supplemental Fig 20: Relevant analyses of the ETSP Station 9 metagenomic depth profile. A) Normalized reads for photosystem II reaction center protein D2 (*psbD)* for the most common photosynthesizers and chlorophyll fluorescence. B) SAR11 RNA polymerase subunit beta (*rpoB*) normalized reads and SAR11 percent of microbial community calculated from *rpoB* normalized reads. C) Proteorhodopsin (*prd*) normalized reads and *prd/rpoB* ratio. D) Derivative of *prd/rpoB* to show where largest changes in the ratio occur in the water column. E) Simpsons Index of Diversity of SAR11 ecotypes based on *rpoB* normalized reads.

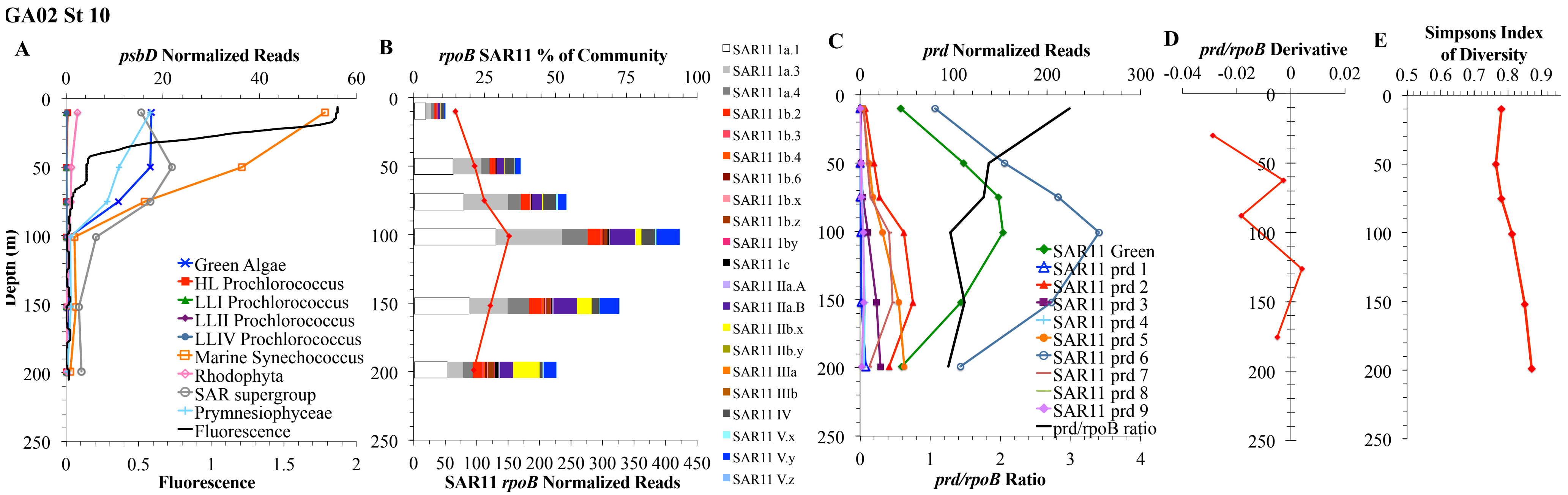

Supplemental Fig 21: Relevant analyses of the Geotraces GA02 Station 10 metagenomic depth profile. A) Normalized reads for photosystem II reaction center protein D2 (*psbD)* for the most common photosynthesizers and chlorophyll fluorescence. B) SAR11 RNA polymerase subunit beta (*rpoB*) normalized reads and SAR11 percent of microbial community calculated from *rpoB* normalized reads. C) Proteorhodopsin (*prd*) normalized reads and *prd/rpoB* ratio. D) Derivative of *prd/rpoB* to show where largest changes in the ratio occur in the water column. E) Simpsons Index of Diversity of SAR11 ecotypes based on *rpoB* normalized reads.

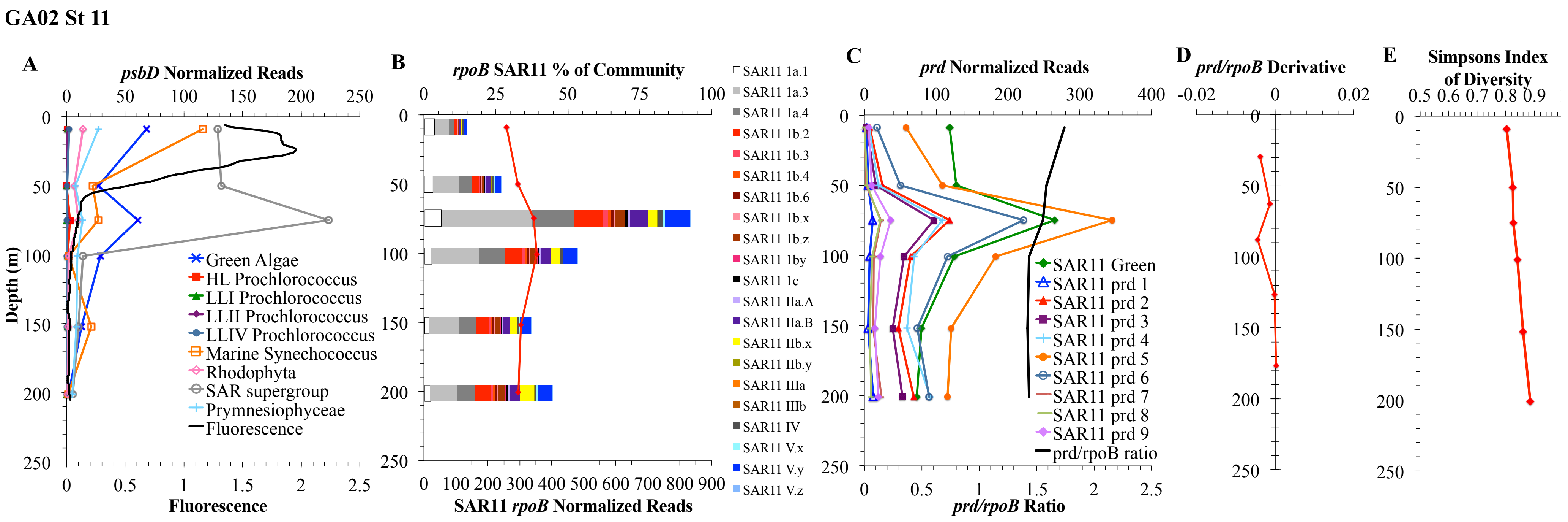

Supplemental Fig 22: Relevant analyses of the Geotraces GA02 Station 11 metagenomic depth profile. A) Normalized reads for photosystem II reaction center protein D2 (*psbD)* for the most common photosynthesizers and chlorophyll fluorescence. B) SAR11 RNA polymerase subunit beta (*rpoB*) normalized reads and SAR11 percent of microbial community calculated from *rpoB* normalized reads. C) Proteorhodopsin (*prd*) normalized reads and *prd/rpoB* ratio. D) Derivative of *prd/rpoB* to show where largest changes in the ratio occur in the water column. E) Simpsons Index of Diversity of SAR11 ecotypes based on *rpoB* normalized reads.

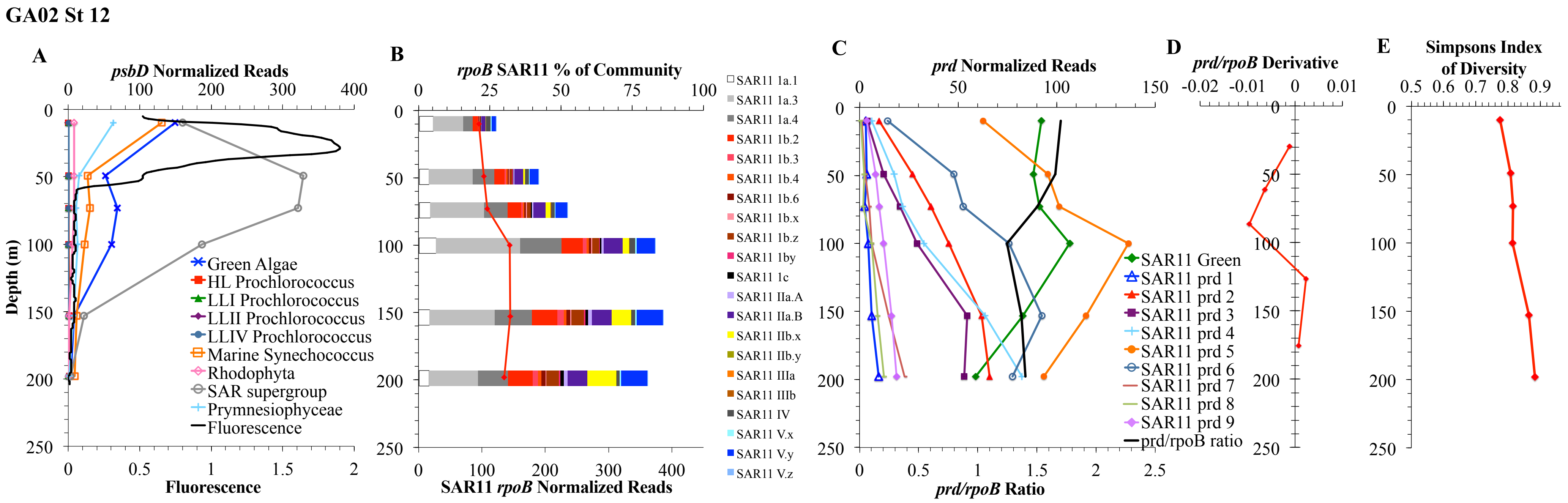

Supplemental Fig 23: Relevant analyses of the Geotraces GA02 Station 12 metagenomic depth profile. A) Normalized reads for photosystem II reaction center protein D2 (*psbD)* for the most common photosynthesizers and chlorophyll fluorescence. B) SAR11 RNA polymerase subunit beta (*rpoB*) normalized reads and SAR11 percent of microbial community calculated from *rpoB* normalized reads. C) Proteorhodopsin (*prd*) normalized reads and *prd/rpoB* ratio. D) Derivative of *prd/rpoB* to show where largest changes in the ratio occur in the water column. E) Simpsons Index of Diversity of SAR11 ecotypes based on *rpoB* normalized reads.

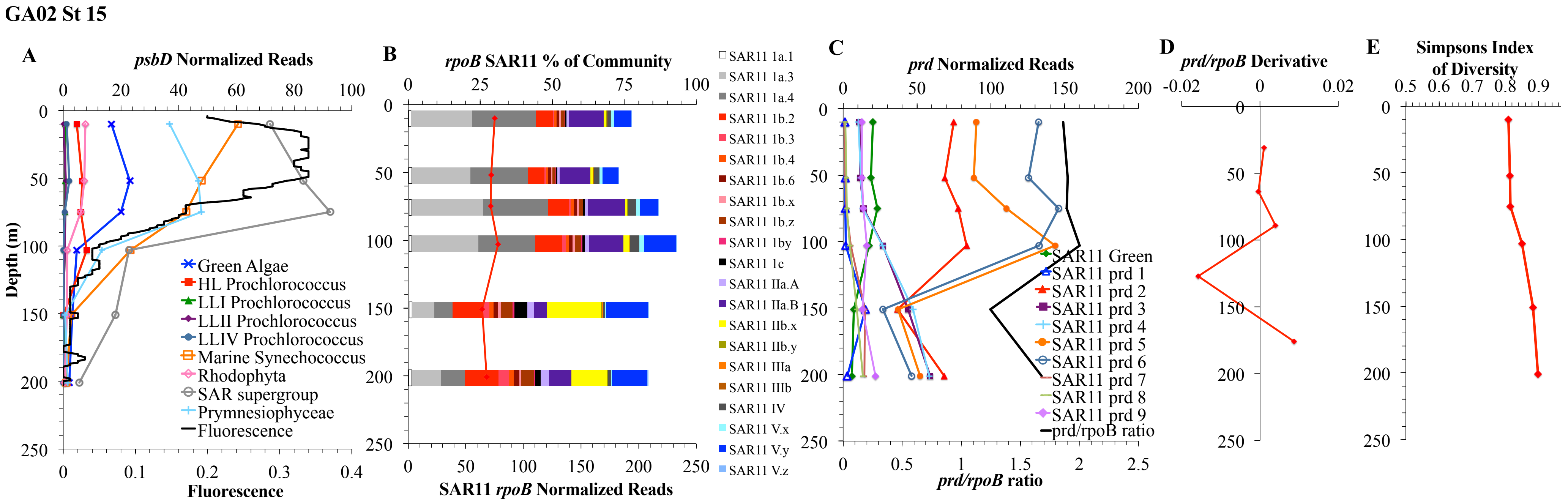

Supplemental Fig 24: Relevant analyses of the Geotraces GA02 Station 15 metagenomic depth profile. A) Normalized reads for photosystem II reaction center protein D2 (*psbD)* for the most common photosynthesizers and chlorophyll fluorescence. B) SAR11 RNA polymerase subunit beta (*rpoB*) normalized reads and SAR11 percent of microbial community calculated from *rpoB* normalized reads. C) Proteorhodopsin (*prd*) normalized reads and *prd/rpoB* ratio. D) Derivative of *prd/rpoB* to show where largest changes in the ratio occur in the water column. E) Simpsons Index of Diversity of SAR11 ecotypes based on *rpoB* normalized reads.

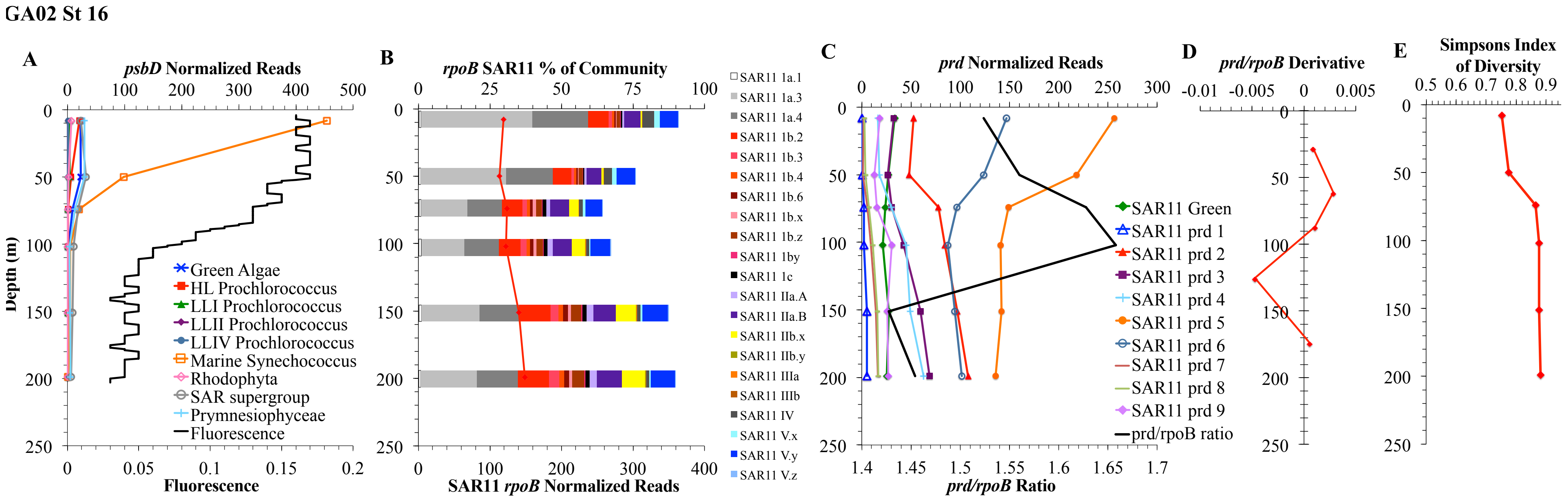

Supplemental Fig 25: Relevant analyses of the Geotraces GA02 Station 16 metagenomic depth profile. A) Normalized reads for photosystem II reaction center protein D2 (*psbD)* for the most common photosynthesizers and chlorophyll fluorescence. B) SAR11 RNA polymerase subunit beta (*rpoB*) normalized reads and SAR11 percent of microbial community calculated from *rpoB* normalized reads. C) Proteorhodopsin (*prd*) normalized reads and *prd/rpoB* ratio. D) Derivative of *prd/rpoB* to show where largest changes in the ratio occur in the water column. E) Simpsons Index of Diversity of SAR11 ecotypes based on *rpoB* normalized reads.

GA02 St 17

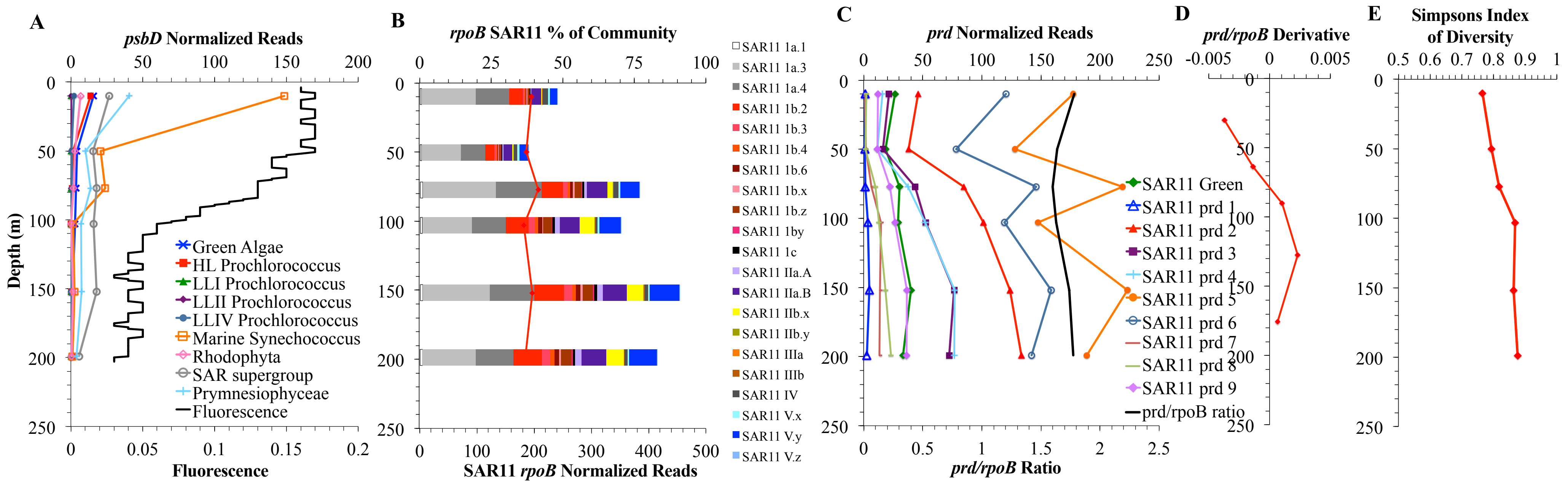

Supplemental Fig 26: Relevant analyses of the Geotraces GA02 Station 17 metagenomic depth profile. A) Normalized reads for photosystem II reaction center protein D2 (*psbD)* for the most common photosynthesizers and chlorophyll fluorescence. B) SAR11 RNA polymerase subunit beta (*rpoB*) normalized reads and SAR11 percent of microbial community calculated from *rpoB* normalized reads. C) Proteorhodopsin (*prd*) normalized reads and *prd/rpoB* ratio. D) Derivative of *prd/rpoB* to show where largest changes in the ratio occur in the water column. E) Simpsons Index of Diversity of SAR11 ecotypes based on *rpoB* normalized reads.

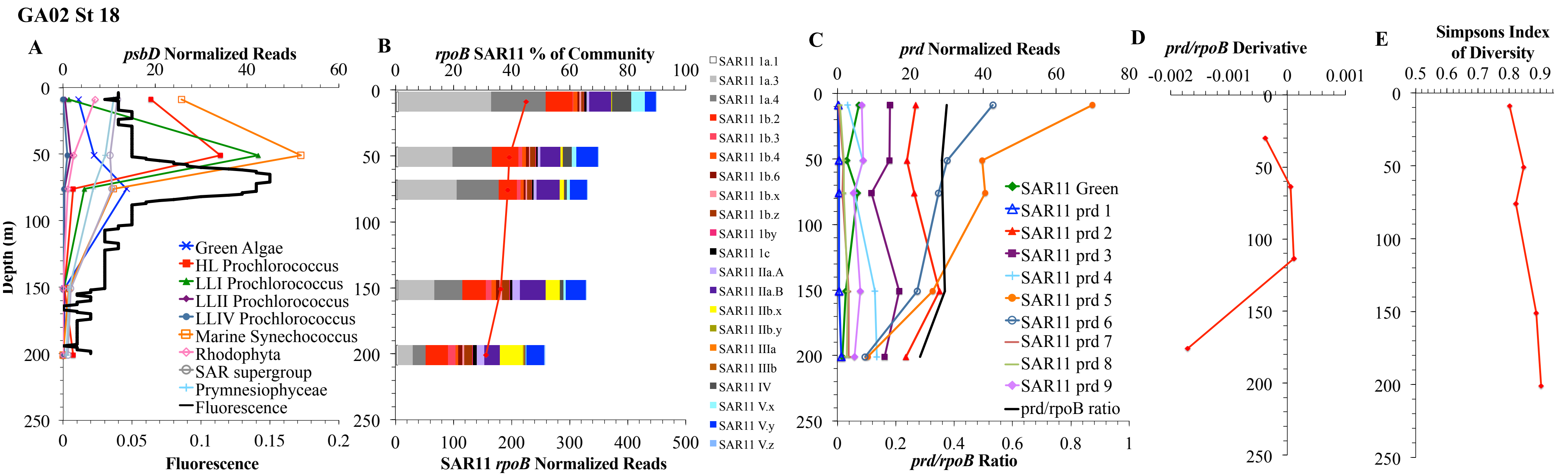

Supplemental Fig 27: Relevant analyses of the Geotraces GA02 Station 18 metagenomic depth profile. A) Normalized reads for photosystem II reaction center protein D2 (*psbD)* for the most common photosynthesizers and chlorophyll fluorescence. B) SAR11 RNA polymerase subunit beta (*rpoB*) normalized reads and SAR11 percent of microbial community calculated from *rpoB* normalized reads. C) Proteorhodopsin (*prd*) normalized reads and *prd/rpoB* ratio. D) Derivative of *prd/rpoB* to show where largest changes in the ratio occur in the water column. E) Simpsons Index of Diversity of SAR11 ecotypes based on *rpoB* normalized reads.

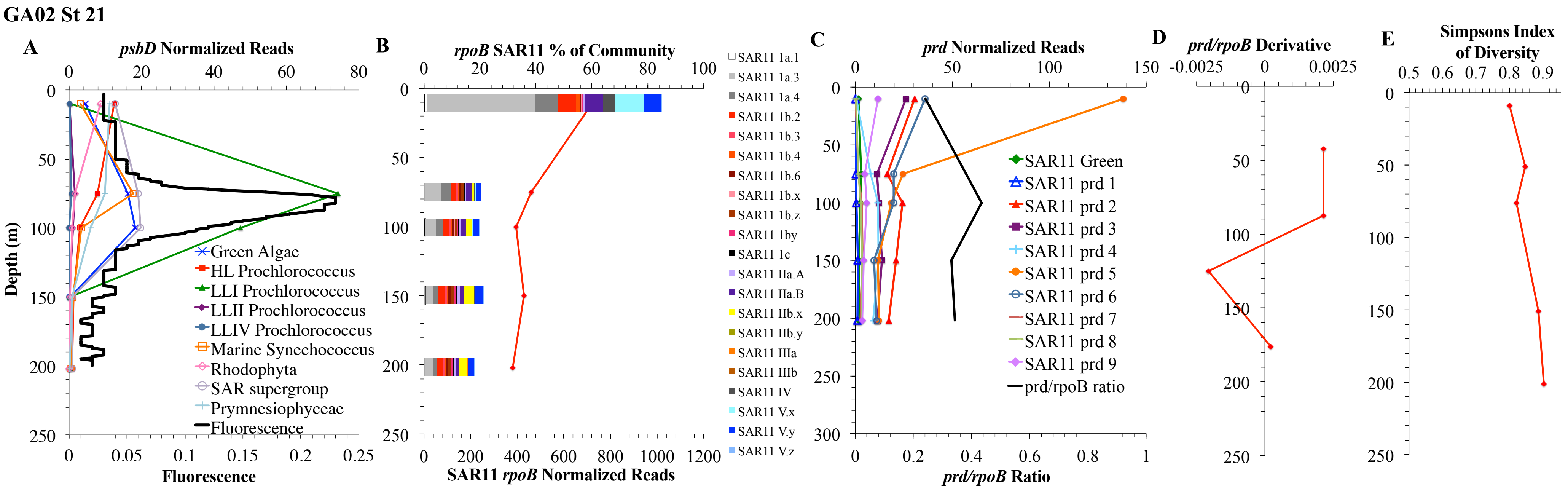

Supplemental Fig 28: Relevant analyses of the Geotraces GA02 Station 21 metagenomic depth profile. A) Normalized reads for photosystem II reaction center protein D2 (*psbD)* for the most common photosynthesizers and chlorophyll fluorescence. B) SAR11 RNA polymerase subunit beta (*rpoB*) normalized reads and SAR11 percent of microbial community calculated from *rpoB* normalized reads. C) Proteorhodopsin (*prd*) normalized reads and *prd/rpoB* ratio. D) Derivative of *prd/rpoB* to show where largest changes in the ratio occur in the water column. E) Simpsons Index of Diversity of SAR11 ecotypes based on *rpoB* normalized reads.

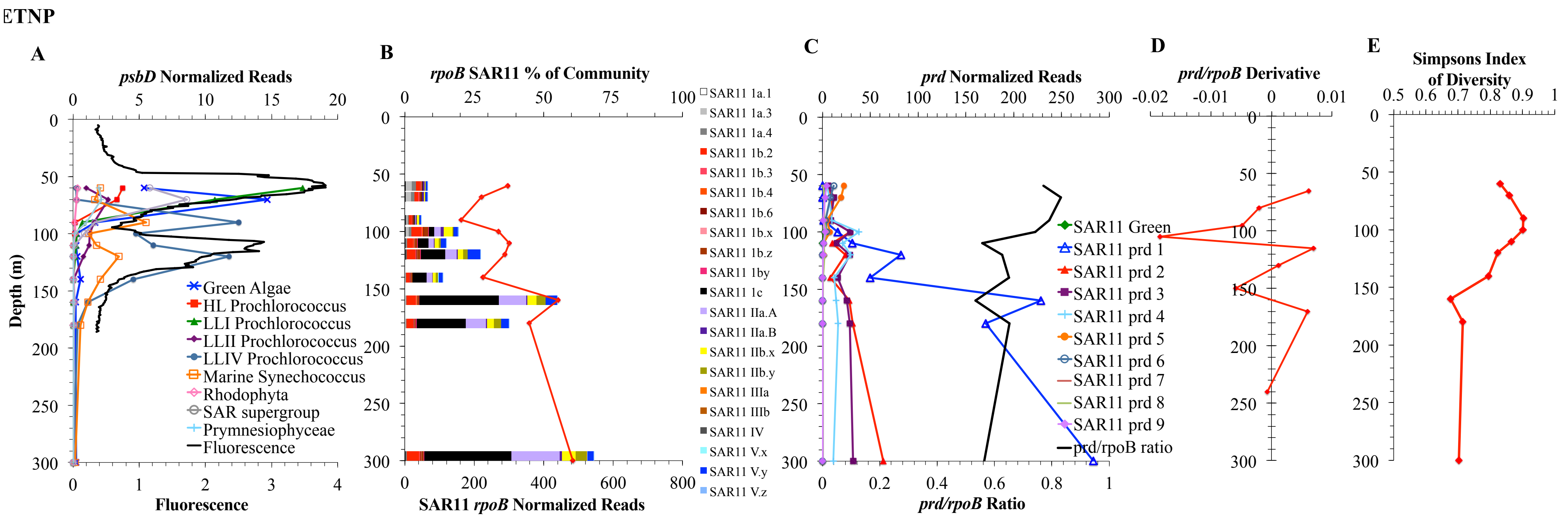

Supplemental Fig 29: Relevant analyses of the ETNP metagenomic depth profile. A) Normalized reads for photosystem II reaction center protein D2 (*psbD)* for the most common photosynthesizers and chlorophyll fluorescence. B) SAR11 RNA polymerase subunit beta (*rpoB*) normalized reads and SAR11 percent of microbial community calculated from *rpoB* normalized reads. C) Proteorhodopsin (*prd*) normalized reads and *prd/rpoB* ratio. D) Derivative of *prd/rpoB* to show where largest changes in the ratio occur in the water column. E) Simpsons Index of Diversity of SAR11 ecotypes based on *rpoB* normalized reads.

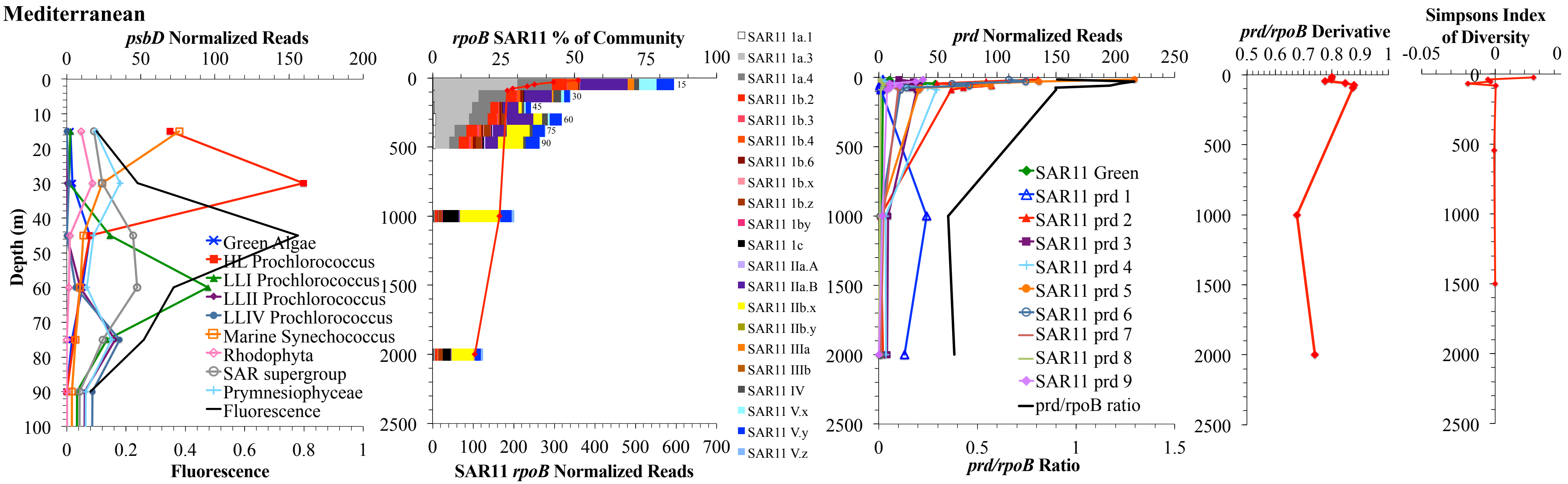

Supplemental Figure 30: Relevant analyses of the Mediterranean metagenomic depth profile showing the top 100 m. A) Normalized reads for photosystem II reaction center protein D2 (*psbD)* for the most common photosynthesizers and chlorophyll fluorescence. B) SAR11 RNA polymerase subunit beta (*rpoB*) normalized reads and SAR11 percent of microbial community calculated from *rpoB* normalized reads. C) Proteorhodopsin (*prd*) normalized reads and *prd/rpoB* ratio. D) Derivative of *prd/rpoB* to show where largest changes in the ratio occur in the water column. E) Simpsons Index of Diversity of SAR11 ecotypes based on *rpoB* normalized reads.

Mediterranean Deep

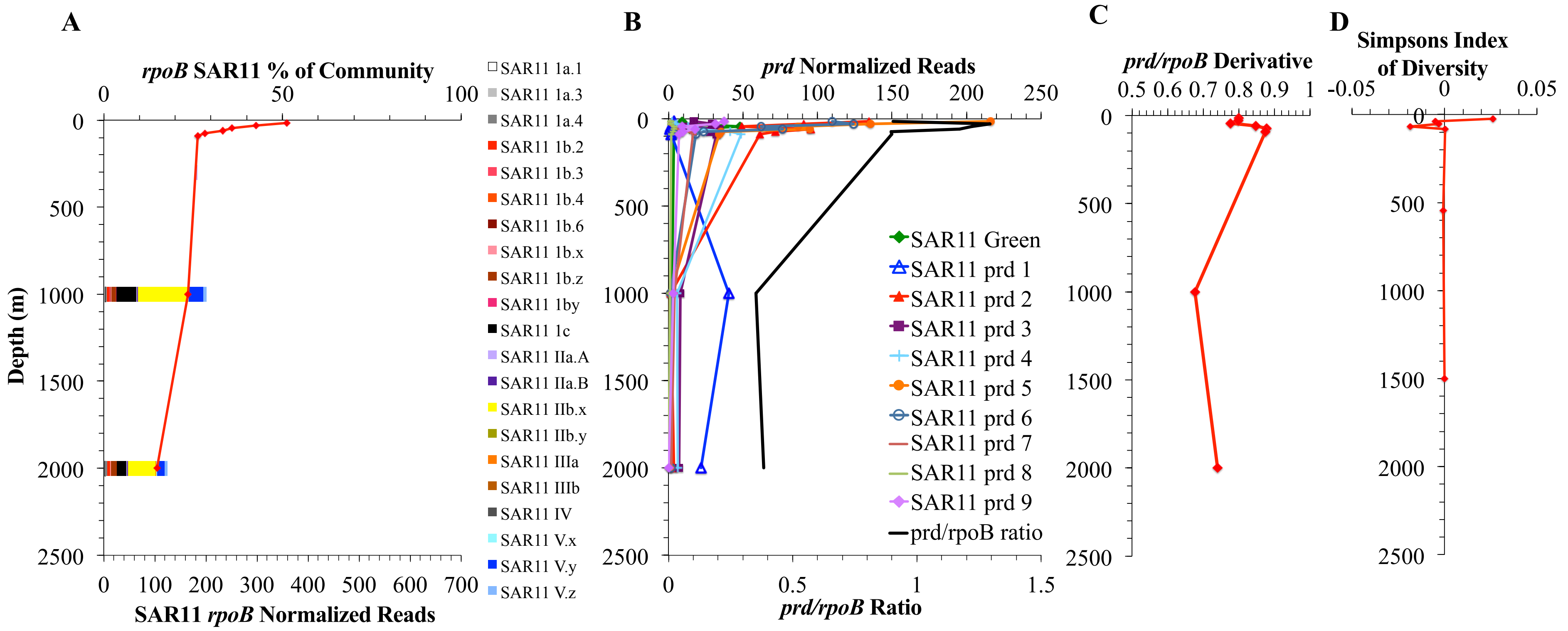

Supplemental Figure 31: Relevant analyses of the Mediterranean metagenomic depth profile showing the entire sampled water column. A) SAR11 RNA polymerase subunit beta (*rpoB*) normalized reads and SAR11 percent of microbial community calculated from *rpoB* normalized reads. B) Proteorhodopsin (*prd*) normalized reads and *prd/rpoB* ratio. C) Derivative of *prd/rpoB* to show where largest changes in the ratio occur in the water column. D) Simpsons Index of Diversity of SAR11 ecotypes based on *rpoB* normalized reads.

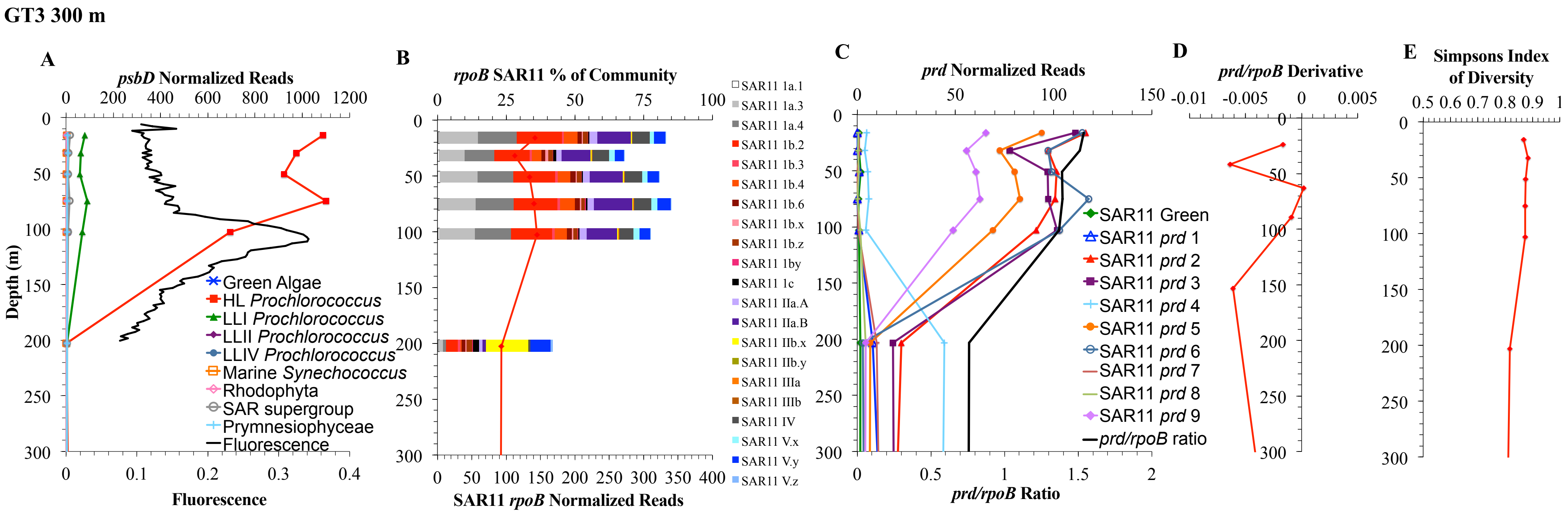

Supplemental Figure 32: Relevant analyses of the Geotraces GP13 cruise Station GT3 metagenomic depth profile showing the top 300 m. A) Normalized reads for photosystem II reaction center protein D2 (*psbD)* for the most common photosynthesizers and chlorophyll fluorescence. B) SAR11 RNA polymerase subunit beta (*rpoB*) normalized reads and SAR11 percent of microbial community calculated from *rpoB* normalized reads. C) Proteorhodopsin (*prd*) normalized reads and *prd/rpoB* ratio. D) Derivative of *prd/rpoB* to show where largest changes in the ratio occur in the water column. E) Simpsons Index of Diversity of SAR11 ecotypes based on *rpoB* normalized reads.

GT3 Deep

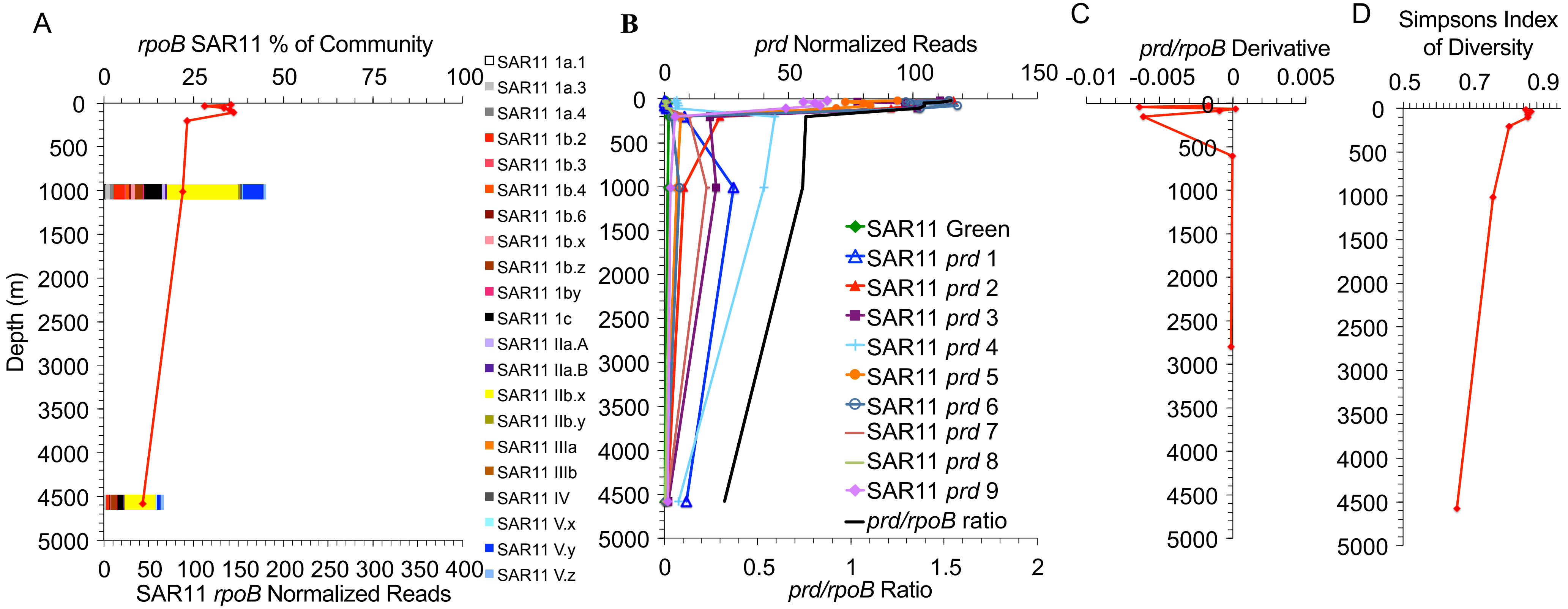

Supplemental Figure 33: Relevant analyses of the Geotraces GP13 cruise Station GT3 metagenomic depth profile showing the entire sampled water column. A) SAR11 RNA polymerase subunit beta (*rpoB*) normalized reads and SAR11 percent of microbial community calculated from *rpoB* normalized reads. B) Proteorhodopsin (*prd*) normalized reads and *prd/rpoB* ratio. C) Derivative of *prd/rpoB* to show where largest changes in the ratio occur in the water column. D) Simpsons Index of Diversity of SAR11 ecotypes based on *rpoB* normalized reads.

GT8 300 m

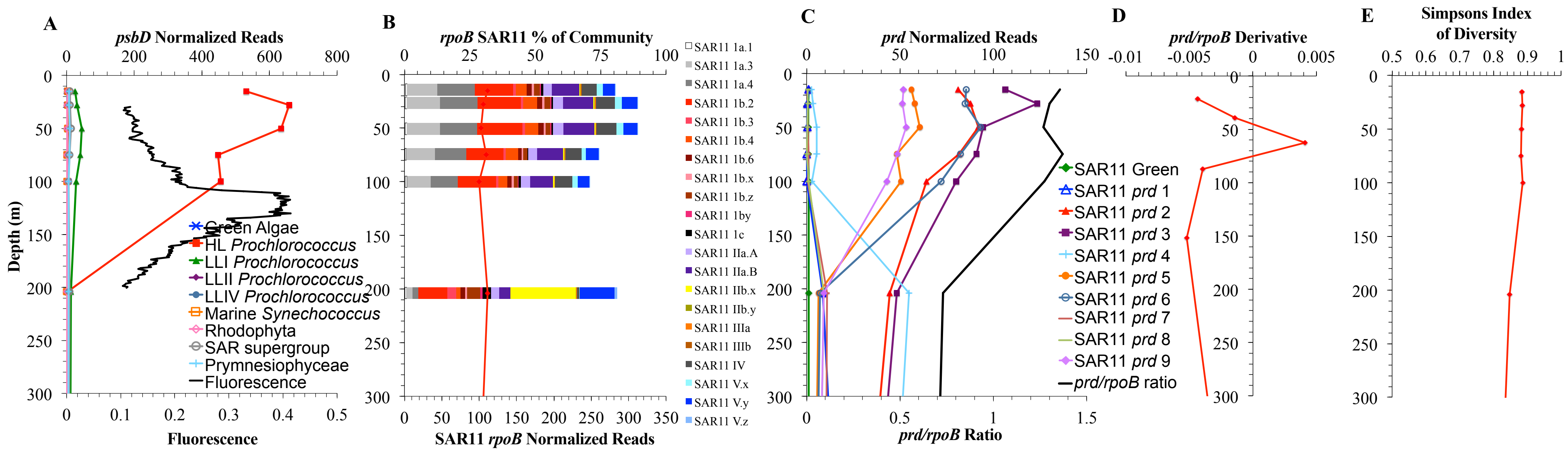

Supplemental Figure 34: Relevant analyses of the Geotraces GP13 cruise Station GT8 metagenomic depth profile showing the top 300 m. A) Normalized reads for photosystem II reaction center protein D2 (*psbD)* for the most common photosynthesizers and chlorophyll fluorescence. B) SAR11 RNA polymerase subunit beta (*rpoB*) normalized reads and SAR11 percent of microbial community calculated from *rpoB* normalized reads. C) Proteorhodopsin (*prd*) normalized reads and *prd/rpoB* ratio. D) Derivative of *prd/rpoB* to show where largest changes in the ratio occur in the water column. E) Simpsons Index of Diversity of SAR11 ecotypes based on *rpoB* normalized reads.

GT8 Deep

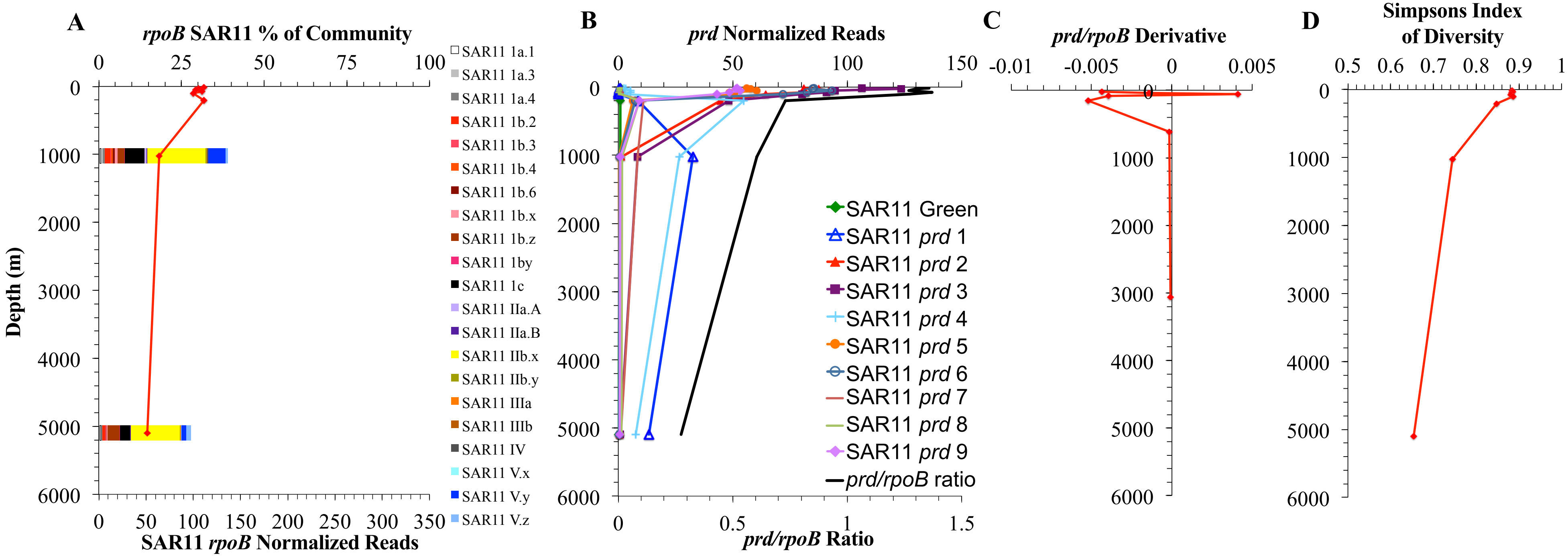

Supplemental Figure 35: Relevant analyses of the Geotraces GP13 cruise Station GT8 metagenomic depth profile showing the entire sampled water column. A) SAR11 RNA polymerase subunit beta (*rpoB*) normalized reads and SAR11 percent of microbial community calculated from *rpoB* normalized reads. B) Proteorhodopsin (*prd*) normalized reads and *prd/rpoB* ratio. C) Derivative of *prd/rpoB* to show where largest changes in the ratio occur in the water column. D) Simpsons Index of Diversity of SAR11 ecotypes based on *rpoB* normalized reads.

GT15 300 m

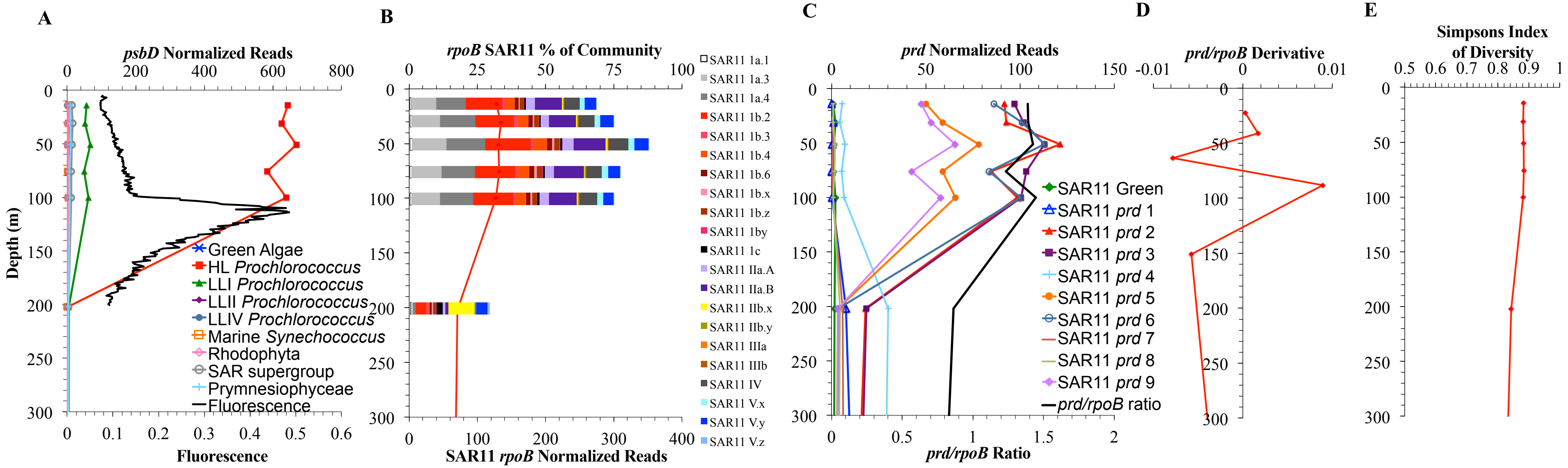

Supplemental Figure 36: Relevant analyses of the Geotraces GP13 cruise Station GT15 metagenomic depth profile showing the top 300 m. A) Normalized reads for photosystem II reaction center protein D2 (*psbD)* for the most common photosynthesizers and chlorophyll fluorescence. B) SAR11 RNA polymerase subunit beta (*rpoB*) normalized reads and SAR11 percent of microbial community calculated from *rpoB* normalized reads. C) Proteorhodopsin (*prd*) normalized reads and *prd/rpoB* ratio. D) Derivative of *prd/rpoB* to show where largest changes in the ratio occur in the water column. E) Simpsons Index of Diversity of SAR11 ecotypes based on *rpoB* normalized reads.

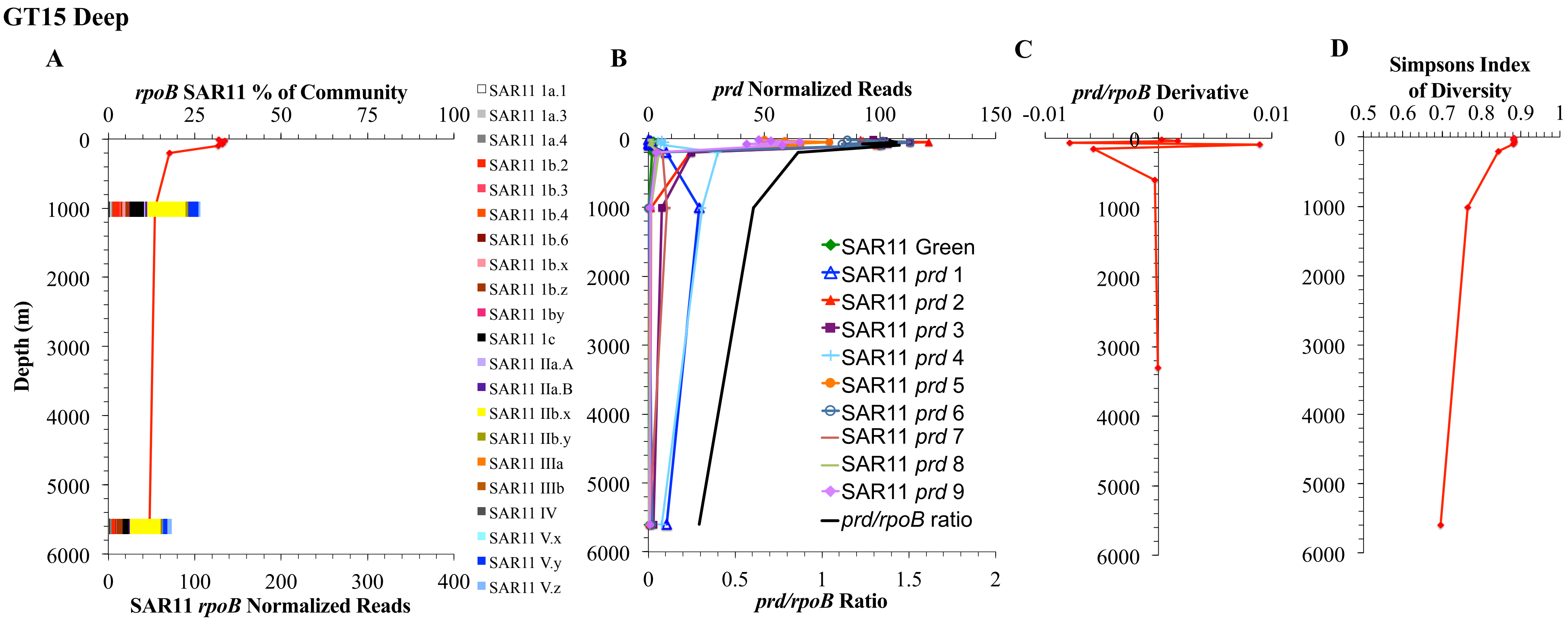

Supplemental Figure 37: Relevant analyses of the Geotraces GP13 cruise Station GT15 metagenomic depth profile showing the entire sampled water column. A) SAR11 RNA polymerase subunit beta (*rpoB*) normalized reads and SAR11 percent of microbial community calculated from *rpoB* normalized reads. B) Proteorhodopsin (*prd*) normalized reads and *prd/rpoB* ratio. C) Derivative of *prd/rpoB* to show where largest changes in the ratio occur in the water column. D) Simpsons Index of Diversity of SAR11 ecotypes based on *rpoB* normalized reads.

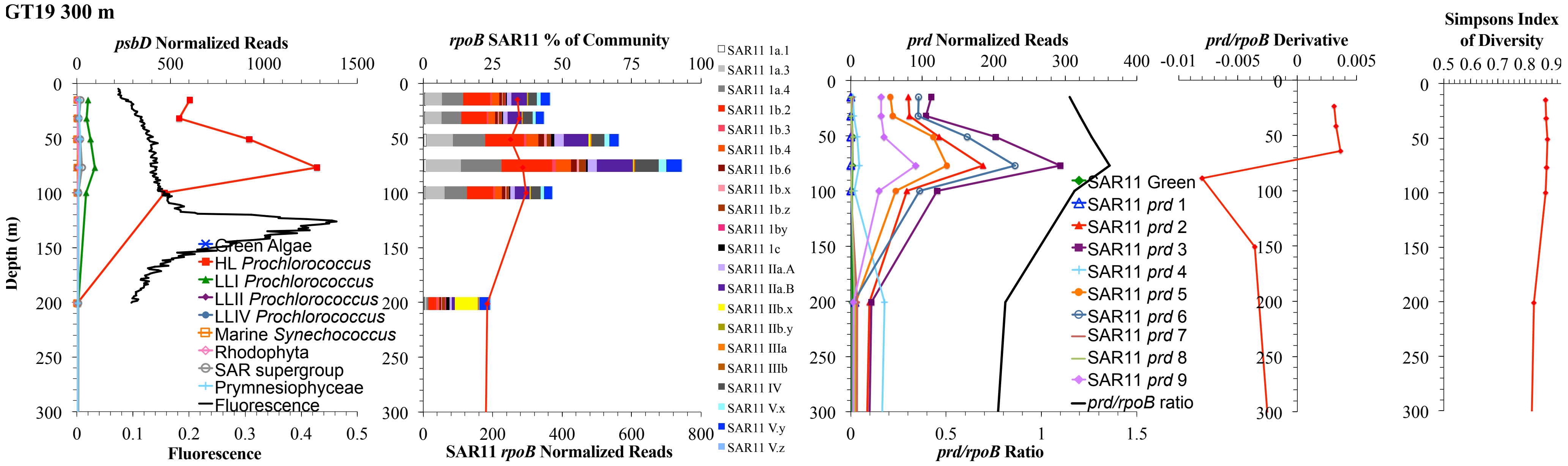

Supplemental Figure 38: Relevant analyses of the Geotraces GP13 cruise Station GT19 metagenomic depth profile showing the top 300 m. A) Normalized reads for photosystem II reaction center protein D2 (*psbD)* for the most common photosynthesizers and chlorophyll fluorescence. B) SAR11 RNA polymerase subunit beta (*rpoB*) normalized reads and SAR11 percent of microbial community calculated from *rpoB* normalized reads. C) Proteorhodopsin (*prd*) normalized reads and *prd/rpoB* ratio. D) Derivative of *prd/rpoB* to show where largest changes in the ratio occur in the water column. E) Simpsons Index of Diversity of SAR11 ecotypes based on *rpoB* normalized reads.

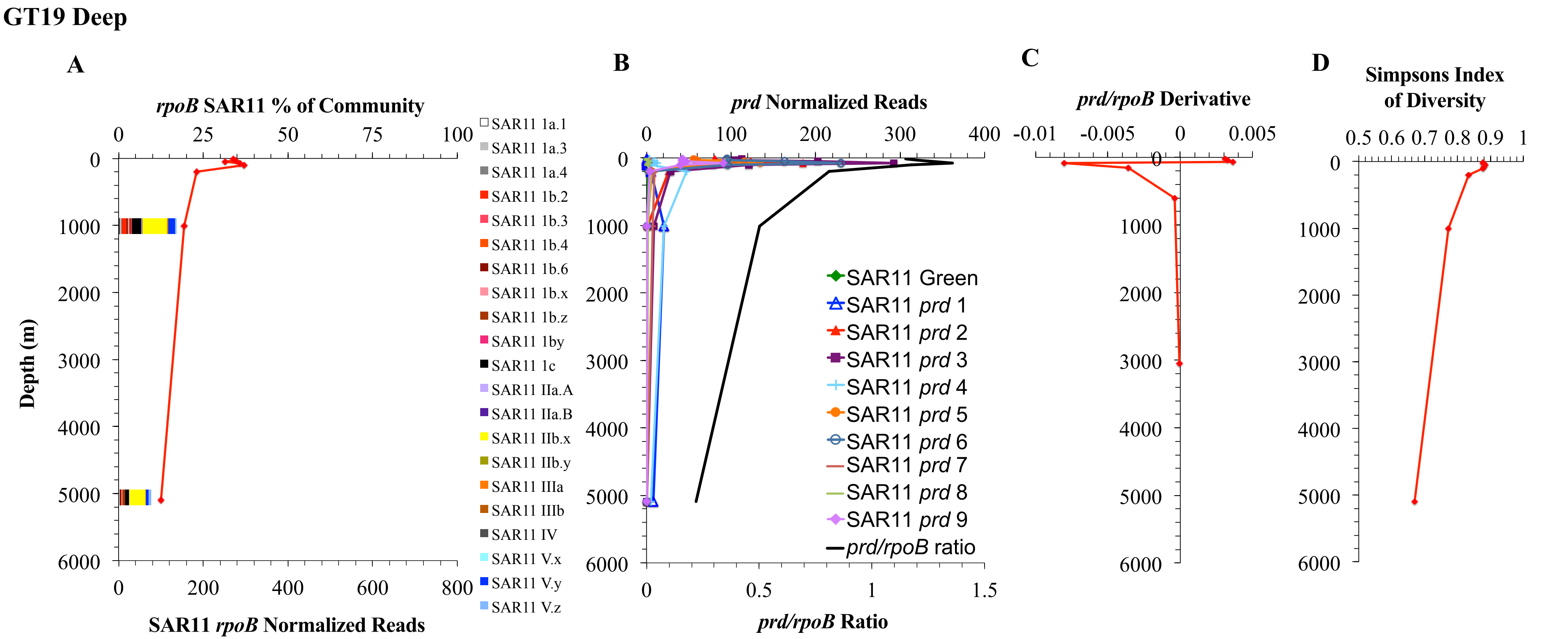

Supplemental Figure 39: Relevant analyses of the Geotraces GP13 cruise Station GT19 metagenomic depth profile showing the entire sampled water column. A) SAR11 RNA polymerase subunit beta (*rpoB*) normalized reads and SAR11 percent of microbial community calculated from *rpoB* normalized reads. B) Proteorhodopsin (*prd*) normalized reads and *prd/rpoB* ratio. C) Derivative of *prd/rpoB* to show where largest changes in the ratio occur in the water column. D) Simpsons Index of Diversity of SAR11 ecotypes based on *rpoB* normalized reads.

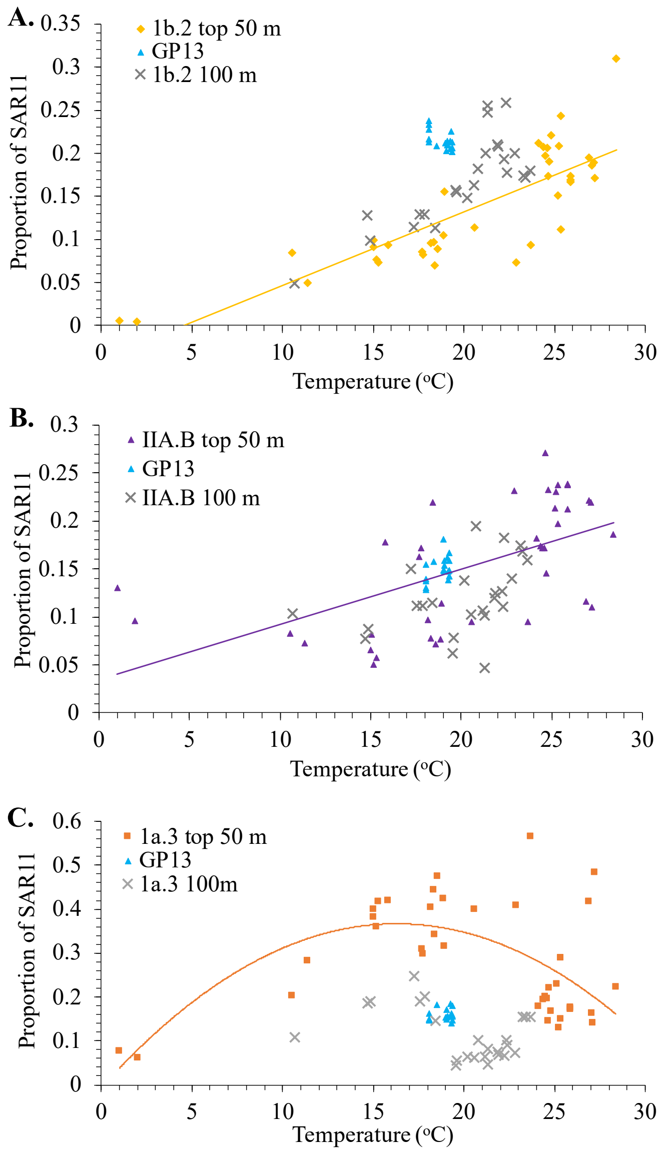

Figure S40. Including data from transect GP13 in an examination of SAR11 ecotypes in the upper water column across our dataset compared to temperature. Ecotype data from the top 50 m was compared to data from 100 m.
