# Supplementary material for: SAR11 ecotypes across ocean basins change with depth due to changes in light and oxygen
Source: ISME J. 2025 Oct 8;19(1):wraf221. doi: 10.1093/ismejo/wraf221 (PMC12598773; doi:10.1093/ismejo/wraf221)
Supplement: Supplemental_table_fig_wraf221 [file Supplemental_figure_1_trees.pdf › Supplemental_table_fig_wraf221/Supplemental_figure_1_trees.pdf]

A

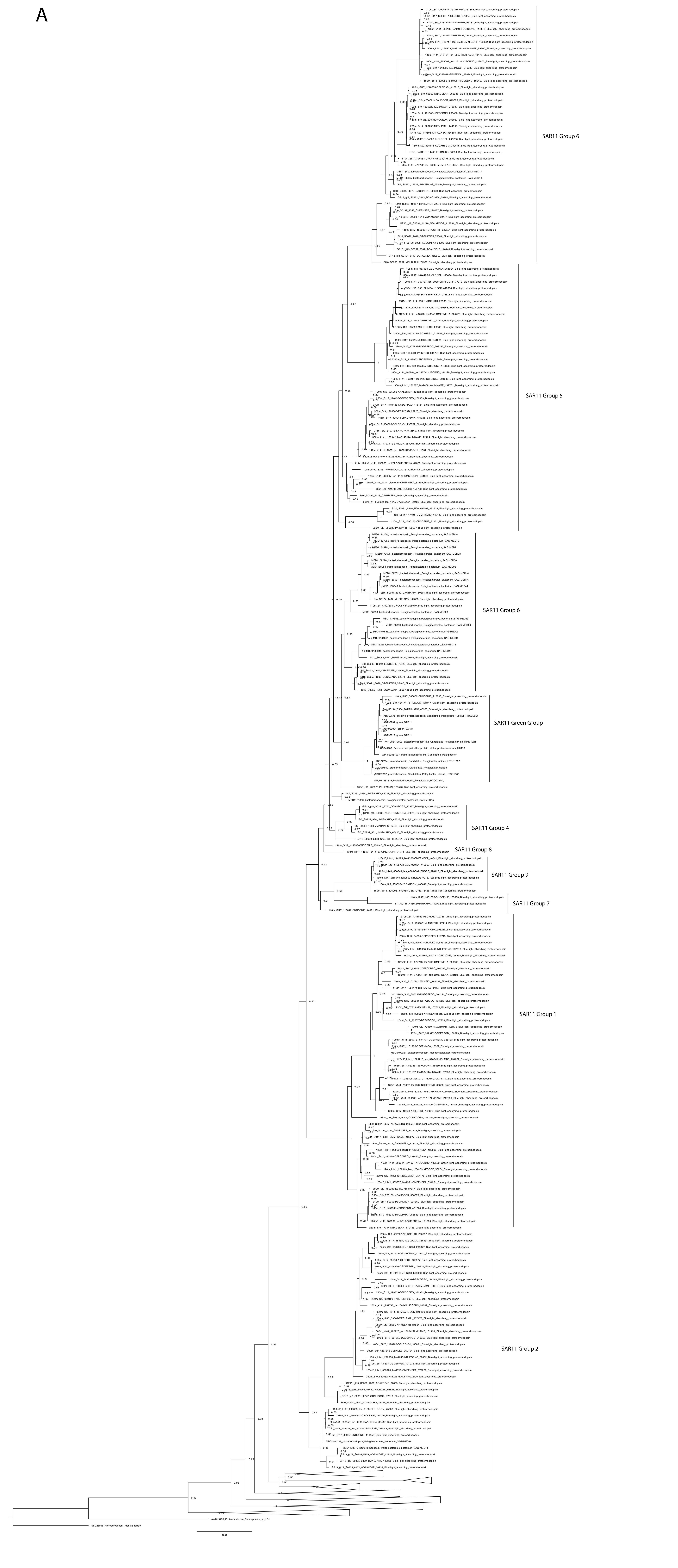

B

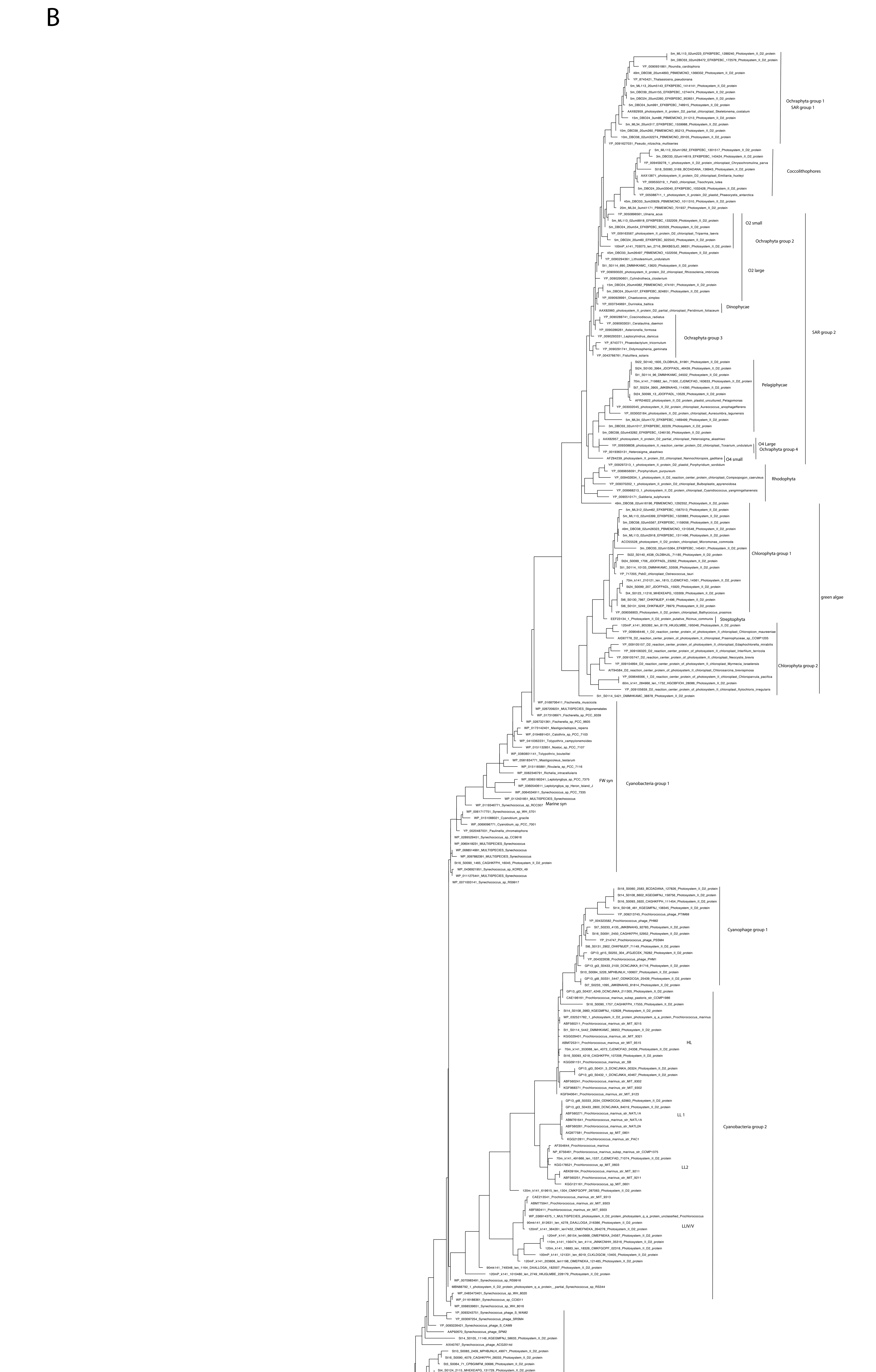

Supplemental Figure 1: A) Phylogenetic tree of the SAR11 Proteorhodopsin *prd* gene. B) Phylogenetic tree of relevant primary producers photosystem II D2 protein *psbD*.
